# High Proton Conduction in the Octahedral Layers of Fully Hydrated Hexagonal Perovskite-Related Oxides

**DOI:** 10.1021/jacs.4c04325

**Published:** 2024-06-25

**Authors:** Kohei Matsuzaki, Kei Saito, Yoichi Ikeda, Yusuke Nambu, Masatomo Yashima

**Affiliations:** † Department of Chemistry, School of Science, Tokyo Institute of Technology, 2-12-1-W4-17, O-okayama, Meguro-ku, Tokyo 152-8551, Japan; ‡ Institute for Materials Research, Tohoku University, 2-1-1 Katahira, Aoba-ku, Sendai 980-8577, Japan; § Organization for Advanced Studies, Tohoku University, 2-1-1 Katahira, Aoba-ku, Sendai 980-8577, Japan; ∥ FOREST, Japan Science and Technology Agency, 4-1-8 Honcho, Kawaguchi, Saitama 332-0012, Japan

## Abstract

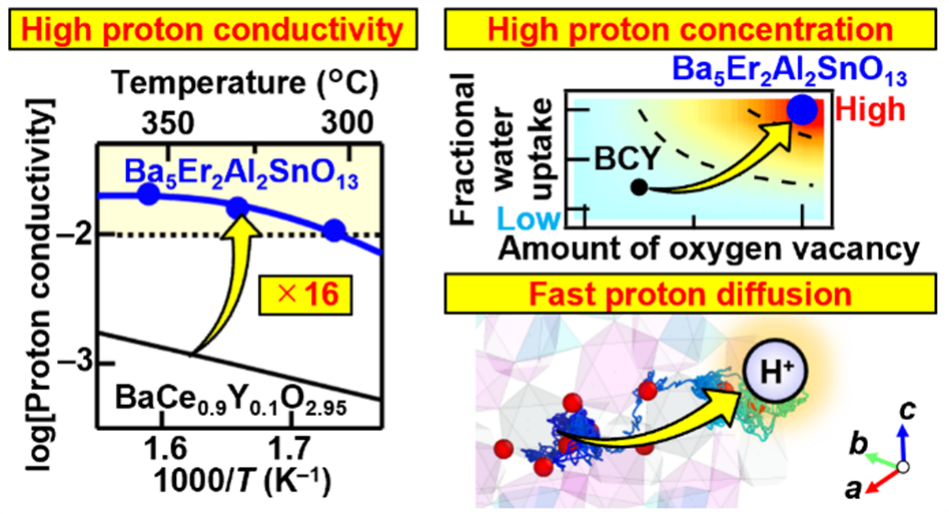

Proton conductors have potential applications such as fuel cells, electrolysis cells, and sensors. These applications require new materials with high proton conductivity and high chemical stability at intermediate temperatures. Herein we report a series of new hexagonal perovskite-related oxides, Ba_5_*R*_2_Al_2_SnO_13_ (*R* = Gd, Dy, Ho, Y, Er, Tm, and Yb). Ba_5_Er_2_Al_2_SnO_13_ exhibited a high proton conductivity without chemical doping (e.g., 0.01 S cm^–1^ at 303 °C), which is attributed to its high proton concentration and diffusion coefficient. The high diffusion coefficient of Ba_5_Er_2_Al_2_SnO_13_ can be attributed to the fast proton migration in the octahedral layers. The high proton concentration is attributed to full hydration in hydrated Ba_5_Er_2_Al_2_SnO_13_ and the large amount of intrinsic oxygen vacancies in the dry sample, as evidenced by both neutron diffraction and thermogravimetric analysis. Ba_5_Er_2_Al_2_SnO_13_ was found to exhibit high chemical stability under wet atmospheres of O_2_, air, H_2_, and CO_2_. High proton conductivity and high chemical stability indicate that Ba_5_Er_2_Al_2_SnO_13_ is a superior proton conductor. Ba_5_*R*_2_Al_2_SnO_13_ (*R* = Gd, Dy, Ho, Y, Tm, and Yb) exhibited high electrical conductivity in wet N_2_, suggesting that these materials also exhibit high proton conductivity. These findings will open new avenues for proton conductors. The high proton conductivity via full hydration and fast proton migration in octahedral layers in highly oxygen-deficient hexagonal perovskite-related materials would be an effective strategy for developing next-generation proton conductors.

## 1 Introduction

Fuel cells are electrochemical devices that directly convert chemical energy from fuel into electrical energy.^1−7^ Solid oxide fuel cells (SOFCs) have advantages such as high energy efficiency and fuel flexibility. Since the SOFCs use oxide ion conductors as electrolytes, the working temperature of SOFCs is high (>700 °C), resulting in high cost and degradation.^8−10^ To solve this problem, protonic ceramic fuel cells (PCFCs) have attracted attention as a technological alternative to SOFCs because proton conductors are known to exhibit higher conductivity at intermediate temperatures (200–500 °C) compared to oxide ion conductors.^11−19^ To develop high-performance PCFCs, it is important to explore novel proton conductors that exhibit both high proton conductivity and high chemical stability. Salts, hydrates, and polymers generally exhibit high proton conductivity but decompose at intermediate temperatures.^3−5^ For example, CsH_2_PO_4_ solid acids exhibit high proton conductivity of over 0.01 S cm^–1^ within a limited temperature range of 230–254 °C.^3^ However, their application is limited due to the decomposition above 254 °C. In contrast, oxides generally exhibit high chemical stability and low proton conductivity at intermediate temperatures. As a result, there are no materials that exhibit both high proton conductivity and high chemical stability at intermediate temperatures,^20,21^ although the lack of suitable materials has stimulated the search for new proton conductors. The purpose of this work is to explore new oxide materials with high proton conductivity and chemical stability.

The hexagonal perovskite-related oxides are a class of perovskite-related materials. The crystal structure of a hexagonal perovskite-related oxide has hexagonal close-packed (h) *A*O_3_ layer(s) and/or intrinsically oxygen-deficient hexagonal close-packed *A*O_3−δ_ (h*′*) layer(s),^22,23^ where *A* is a large cation such as Ba^2+^ and δ is the amount of oxygen vacancies. Many hexagonal perovskite-related oxides also contain cubic close-packed (c) and/or anion-deficient cubic close-packed (c′) layers. Recently, the hexagonal perovskite-related oxides have been reported to exhibit significant proton conduction without chemical doping due to the presence of intrinsic oxygen vacancies.^6,13,24−35^ Here, the intrinsic oxygen vacancies are the oxygen vacancies in a parent material. However, the proton conduction of hexagonal perovskite-related oxides has not been extensively studied compared to that in *AB*O_3−δ_ perovskite oxides.^36−38^ Murakami et al.^28^ reported Ba_5_Er_2_Al_2_ZrO_13_ as a new class of proton conductors with high proton conductivity comparable to those of representative proton conductors (e.g., 10^–3^ S cm^–1^ at 300 °C). They refined the crystal structure of hydrated Ba_5_Er_2_Al_2_ZrO_13_ using neutron diffraction data. Ba_5_Er_2_Al_2_ZrO_13_ is a hexagonal perovskite-related oxide with h*′* layers and octahedral layers. Here, the octahedral layer is the [ErO_6_–ZrO_6_–ErO_6_] triple octahedral layer, which consists of two ErO_6_ octahedral layers and one ZrO_6_ octahedral layer and can be regarded as a triple perovskite-like layer. Murakami et al.^28^ proposed the water uptake leading to the formation of protons in the h*′* layers, which undergo two-dimensional proton diffusion. Later, Youn et al.^33^ also showed the anisotropic proton migration in Ba_5_Er_2_Al_2_ZrO_13_ experimentally and by density functional theory (DFT) calculations. Ba_5_*A*_2_Al_2_ZrO_13_-based oxides (*A* = Dy, Tm, Yb, Lu, In, Y_1/4_Ho_1/4_Er_1/4_Yb_1/4_, Y_1/5_Dy_1/5_Ho_1/5_Er_1/5_Yb_1/5_, and Y_0.5_In_1.5_) have the same crystal structure as Ba_5_Er_2_Al_2_ZrO_13_ (10H polytype, hexagonal crystal system, space group *P*6_3_/*mmc*) and also exhibit significant proton conductivity.^26,28,30,32,33^

In general, a high proton concentration *y* is required for high proton conductivity. Here, the *y* values of hydrated perovskite-type Ba*B*O_3−δ_ and hexagonal perovskite-related oxide Ba_5_*A*_2_Al_2_ZrO_13_ (*A* = rare earth, In) are defined by the chemical formula Ba*B*O_3−δ+*y*/2_H_*y*_ and Ba_5_*A*_2_Al_2_ZrO_13+5*y*/2_H_5*y*_ = 5Ba(*A*_2/5_Al_2/5_Zr_1/5_)O_2.6+*y*/2_H_*y*_, respectively, and *y* is the number of protons per Ba atom. The proton concentrations of Ba_5_*A*_2_Al_2_ZrO_13_ materials are not high due to partial hydration.^26,28,30^ For example, the proton concentration *y* in Ba_5_Er_2_Al_2_ZrO_13–5*y*/2_(OH)_5*y*_ is 0.092 (ref (28)), which is lower than the theoretical proton concentration for full hydration (*y* = 0.4), as shown later. The proton concentration of fully hydrated hexagonal perovskite-related oxides will be higher compared to that of partially hydrated ones; thus, full hydration will result in higher proton conductivity. However, to the best of our knowledge, there are no reports of fully hydrated hexagonal perovskite-related materials. In addition, to the best of our knowledge, there are no reports on Ba_5_*A*_2_Al_2_*M*O_13_ materials (*A* = La, Bi, Nd, Sm, Eu, Gd, Tb, Dy, Ho, Er, Tm, Yb, Lu, Sc, In, and Ga; *M* = Sn, Hf, Ce, Ge, and Ti) in the literature. Therefore, we searched for Ba_5_*A*_2_Al_2_*M*O_13_ materials. In this work, we discovered new materials Ba_5_*R*_2_Al_2_SnO_13_ (*R* = Gd, Dy, Ho, Y, Er, Tm, and Yb). Among them, Ba_5_Er_2_Al_2_SnO_13_ (BEAS) was found to exhibit full hydration and high chemical stability. The full hydration of BEAS enables high proton concentration and conductivity (e.g., 0.01 S cm^–1^ at 303 °C, which is higher than those of other proton conductors).

## 2 Results and Discussion

The new material Ba_5_Er_2_Al_2_SnO_13–5*y*/2_(OH)_5*y*_ (BEAS) (= Ba_5_Er_2_Al_2_SnO_13_·(5*y*/2)H_2_O = Ba_5_Er_2_Al_2_SnO_13–5*y*/2_(OH)_5*y*_□_1–5*y*/2_) where *y* and □ are the proton concentration (0 ≤ *y* ≤ 0.4) and intrinsic oxygen vacancies, respectively, was synthesized by solid-state reactions. All reflections of the Cu Kα X-ray powder diffraction (XRD) pattern of the as-prepared BEAS powders were indexed to the primitive hexagonal lattice (Figure S1), indicating a single hexagonal phase with a 10H polytype. X-ray photoelectron spectroscopy (XPS) data of BEAS indicated that the valences of Ba, Er, Al, and Sn atoms were +2, +3, +3, and +4, respectively (Figure S2).

**Figure 1:**
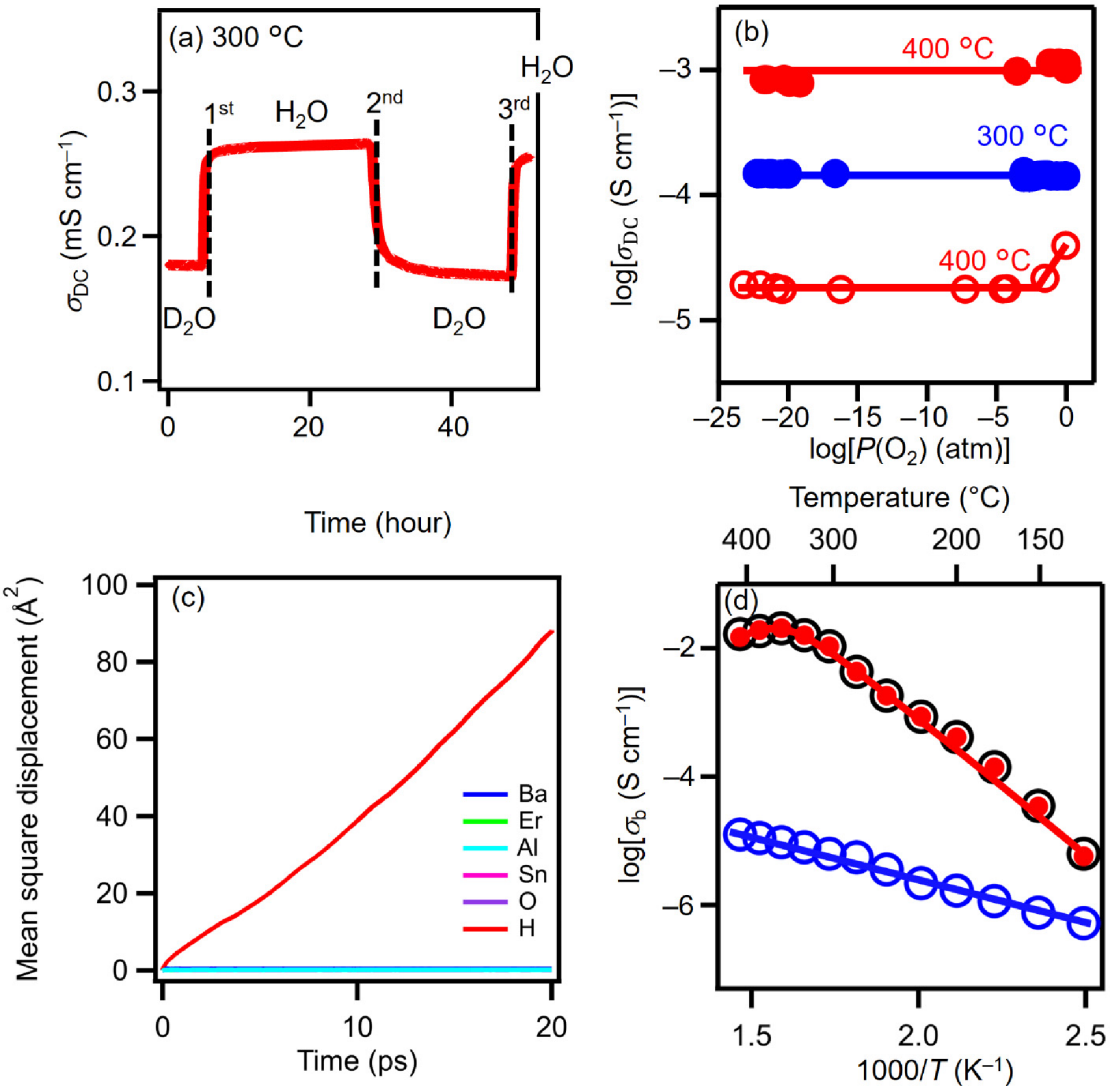
Evidence of the proton conduction in Ba_5_Er_2_Al_2_SnO_13_ (BEAS). (a) H/D isotope effect of the DC electrical conductivity σ_DC_ of BEAS at 300 °C (vapor partial pressure of 0.021 atm). (b) Oxygen partial pressure dependencies of σ_DC_ of BEAS at 300 °C in wet atmospheres (blue solid circles and line) and at 400 °C in wet atmospheres (red solid circles and line) and dry atmospheres (red open circles and line). (c) Mean square displacement (MSD) of atoms obtained by ab initio molecular dynamics simulation at 1200 °C. (d) Arrhenius plots of bulk proton conductivity σ_b_(H^+^) (red closed circles and line) and bulk conductivity in wet N_2_ σ_b_(H_2_O) (black open circles) and dry N_2_ σ_b_(dry) (blue open circles and line). The σ_b_(H^+^) was calculated by the equation: σ_b_(H^+^) = σ_b_(H_2_O) – σ_b_(dry).

To demonstrate the proton conduction of BEAS, H/D isotope exchange experiments were performed at 300 °C in D_2_O-saturated N_2_ and H_2_O-saturated N_2_ (vapor partial pressure of 0.021 atm) (Figure 1a). The atmosphere was switched from D_2_O-saturated N_2_ to H_2_O-saturated N_2_ (first switching in Figure 1a), from H_2_O-saturated N_2_ to D_2_O-saturated N_2_ (second switching in Figure 1a), and then from D_2_O-saturated N_2_ to H_2_O-saturated N_2_ (third switching in Figure 1a). The conductivity ratios σ_DC_(H_2_O)/σ_DC_(D_2_O) at the first, second, and third switchings were approximately 1.5, which is close to 1.41, as expected from the classical theory.^39^ Here, σ_DC_(H_2_O) and σ_DC_(D_2_O) stand for the direct current (DC) electrical conductivities in H_2_O-saturated N_2_ and D_2_O-saturated N_2_, respectively, which were measured by the DC four-probe method. The σ_DC_ of BEAS in wet atmospheres was almost independent of the oxygen partial pressure *P*(O_2_) and had a constant value in the wide *P*(O_2_) ranges from 2 × 10^–22^ to 1 atm at 400 and 600 °C and from 6 × 10^–23^ to 1 atm at 300 °C, indicating negligible electronic conduction and high chemical and electrical stability (Figures 1b and S4). In contrast, in dry atmospheres, the slope of log(σ_DC_) versus log(*P*(O_2_)) of BEAS had a positive value in the high *P*(O_2_) ranges from 3 × 10^–4^ to 1 atm at 600 °C and 3 × 10^–2^ to 1 atm at 400 °C, suggesting hole conduction. At low *P*(O_2_) ranges from 1.6 × 10^–23^ to 3 × 10^–4^ atm in dry atmospheres at 600 °C and 6.6 × 10^–24^ to 5.5 × 10^–5^ atm at 400 °C, the σ_DC_ of BEAS was almost independent of the *P*(O_2_), showing a constant σ_DC_ value, electrolyte domain, negligible electronic conduction, and high chemical and electrical stability. The constant σ_DC_ values independent of *P*(O_2_) can be regarded as ionic conductivities. The proton transport number in wet N_2_ was close to unity between 350 and 500 °C (Figure S3). The proton transport number of BEAS was investigated by electromotive force (EMF) measurements using vapor and oxygen concentration cells and was estimated to be 1.0 at 500 °C. The mean square displacement (MSD) was obtained from ab initio molecular dynamics (AIMD) simulations. The MSD of protons was much higher than the MSDs of other constituents (Figure 1c). These results indicate that the dominant carrier in BEAS is the proton.

Impedance measurements of BEAS were performed to investigate its bulk conductivity σ_b_. To extract the σ_b_ and grain boundary conductivity σ_gb_ of BEAS, equivalent circuit analyses were performed (Figures S5–S9). The obtained bulk capacitance (0.75–7.2 × 10^–12^ F/cm) and grain boundary capacitance 0.95–4.4 × 10^–11^ F/cm were consistent with the values in the literature (Table S1).^40^ The difference between the activation energies for bulk conductivity in D_2_O-saturated N_2_ and H_2_O-saturated N_2_ (*E*_D_ – *E*_H_) was 0.058(5) eV (Table S2), which was close to the *E*_D_ – *E*_H_ value of 0.055 eV predicted by the semiclassical theory.^39^ Here, *E*_D_ and *E*_H_ are the activation energies for the bulk conductivity in D_2_O-saturated N_2_ and H_2_O-saturated N_2_, respectively. The ratio of the pre-exponential factors *A*_H_/*A*_D_ was 0.49, which was consistent with the ratios of other proton conductors.^36,39^ Here, *A*_H_ and *A*_D_ are the pre-exponential factors in H_2_O-saturated N_2_ and D_2_O-saturated N_2_, respectively. The σ_b_ in wet N_2_ was much higher than that in dry N_2_ in the temperature range from 409 to 128 °C (e.g., 2100 times higher at 356 °C; Figure 1d). The proton transport number in wet N_2_ was close to unity between 151 and 409 °C (Figure S10). These results also indicate the proton conduction of BEAS in wet N_2_.

**Figure 2:**
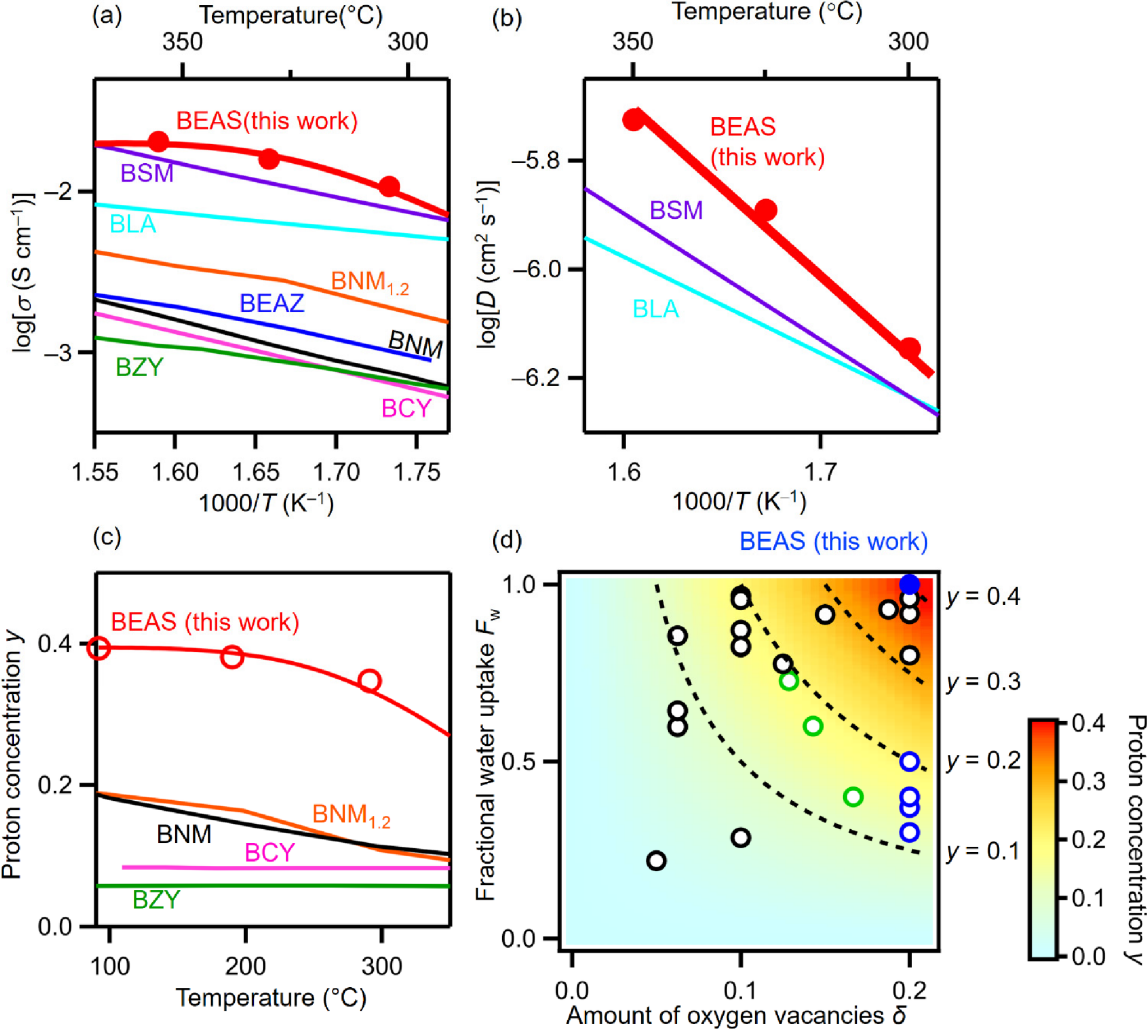
High proton conductivity and its origins of Ba_5_Er_2_Al_2_SnO_13_ (BEAS). (a) Arrhenius plots of the bulk conductivity σ of BEAS in wet N_2_, Ba_2_LuAlO_5_ (BLA) in wet N_2_ (ref (34)), BaSc_0.8_Mo_0.2_O_2.8_ (BSM) in wet air (ref (36)), BaZr_0.8_Y_0.2_O_2.9_ (BZY) in wet air (ref (41)), BaCe_0.9_Y_0.1_O_2.95_ (BCY) in wet air (ref (42)), Ba_7_Nb_3.8_Mo_1.2_O_20.1_ (BNM_1.2_) in wet air (ref (35)), and Ba_7_Nb_4_MoO_20_ (BNM) in wet air (ref (13)) and DC electrical conductivity in wet air σ of Ba_5_Er_2_Al_2_ZrO_13_ (BEAZ) (ref (28)). (b) Arrhenius plots of bulk proton diffusion coefficient *D* obtained using the bulk conductivities and proton concentrations of BEAS, BSM (ref (36)), and BLA (ref (34)). (c) Temperature dependence of proton concentrations in BEAS, BZY (ref (41)), BCY (ref (42)), BNM (ref (13)), and BNM_1.2_ (ref (35)). (d) Plots of the fractional water uptake *F*_w_ (= *y*/2δ) in hydrated perovskite-type and hexagonal perovskite-related oxides against the amount of oxygen vacancies δ in dry materials, showing the proton concentration *y* of hydrated materials. The blue solid circle stands for the data of BEAS, and black, green, and blue open circles denote the data of Ba*B*O_3−δ_·(*y*/2)H_2_O perovskites,^36,43−45^*B*-site-deficient hexagonal perovskite-related materials with the intrinsically oxygen-deficient cubic close-packed c′ layer Ba*B*_1–*x*_O_3−δ_·(*y*/2)H_2_O (*x* = 1/3, 2/7),^13,35^ and Ba*B*O_2.8−δ_·(*y*/2)H_2_O hexagonal perovskite-related materials with h′ layer, respectively.^25,26,30^ Here, *B* cations are relatively smaller than Ba^2+^.

The bulk conductivity of BEAS in wet N_2_ σ_b_(H_2_O) was high (e.g., 0.01 S cm^–1^ at 303 °C in Figure 2a). At 300 °C, BEAS exhibited higher σ_b_(H_2_O) than other proton-conducting perovskite-type and hexagonal perovskite-related oxides^13,34,36,41^ (see the details in Supplementary Note 1). The high σ_b_(H_2_O) of BEAS is attributed to both the high concentration and diffusion coefficient of protons, as discussed later.

The chemical stability of BEAS was investigated by annealing at 600 °C under wet atmospheres of O_2_, H_2_, air, and CO_2_. There was no significant difference between the XRD patterns before and after annealing (Figures S11 and S12), indicating the high chemical stability of BEAS. The high proton conductivity, high proton transport number, high chemical stability, and high chemical and electrical stability indicate that BEAS is a superior proton conductor.

To discuss the origin of the high bulk proton conductivity σ_b_(H^+^) of BEAS, we investigated the bulk proton concentration *y* and the proton diffusion coefficient *D,* because σ_b_(H^+^) is proportional to *y* and *D*: σ_b_(H^+^) ∝ *y* × *D* (Supplementary Note 2). The high *y* and *D* are the origins of the high σ_b_(H^+^) of BEAS (Figure 2b–d), as discussed below. To estimate the *y* of BEAS, we performed thermogravimetric (TG) and thermogravimetric–mass spectrometry (TG-MS) measurements. The TG-MS results indicated that the weight loss on heating was mainly due to dehydration (loss of H_2_O from the BEAS powders) (Figure S13). The TG results of BEAS showed the typical hydration behavior with higher proton concentration at lower temperatures (Figure S14 and Supplementary Note 3). At 300 °C, the proton concentration of BEAS (*y* = 0.35) was higher than those of BaZr_0.8_Y_0.2_O_2.9–*y*/2_(OH)_*y*_ (*y* = 0.06), BaCe_0.9_Y_0.1_O_2.95–*y*/2_(OH)_*y*_ (*y* = 0.08), Ba_7_Nb_4_MoO_20–7*y*/2_(OH)_7*y*_ (= Ba(Nb_4/7_Mo_1/7_)O_20/7–*y*/2_(OH)_*y*_) (*y* = 0.11), and Ba_7_Nb_3.8_Mo_1.2_O_20.1–7*y*/2_(OH)_7*y*_ (= Ba(Nb_3.8/7_Mo_1.2/7_)O_20.1/7–*y*/2_(OH)_*y*_) (*y* = 0.11) (Figure 2c). The higher proton concentration of BEAS is a source of higher proton conductivity compared to other materials. The proton concentration *y* of hydrated perovskite-type and hexagonal perovskite-related oxides increases with an increase in the amount of oxygen vacancies δ of dry materials (Figures S15a and 2d). The value of *y* also increases with increasing fractional water uptake *F*_w_ (= *y*/2δ) (Figures S15b and 2d). Therefore, the higher proton concentration *y* in BEAS (= Ba_5_Er_2_Al_2_SnO_14–5δ_*·*(5*y*/2)H_2_O = Ba_5_(Er_2/5_Al_2/5_Sn_1/5_)_5_O_(14/5–5δ)5_*·*(5*y*/2)H_2_O = 5 Ba(Er_0.4_Sn_0.4_Al_0.2_)O_2.8−δ_*·*(*y*/2)H_2_O) is attributed to the larger amount of oxygen vacancies (δ = 0.2) in dry BEAS without water and higher *F*_w_ of 1.0 in hydrated BEAS compared to other perovskites and perovskite derivatives^13,35,41−44^ (see the details in Supplementary Note 4). These results indicate that the high proton concentration *y* due to the large amount of oxygen vacancies in BEAS without water and the high fractional water uptake (full hydration) is the origin of the high proton conductivity in BEAS. Ba_2_In_2_O_6–2δ_·*y*H_2_O^45,46^ and doped barium zirconates and scandate such as BaZr_0.95_Y_0.05_O_3−δ_·(*y*/2)H_2_O,^43^ BaZr_0.8_In_0.2_O_3−δ_·(*y*/2)H_2_O,^43^ and BaSc_0.8_W_0.2_O_3−δ_·(*y*/2)H_2_O^47^ exhibit full hydration (*F*_w_ = 1.0), as well as BEAS (Figure S16).

**Figure 3:**
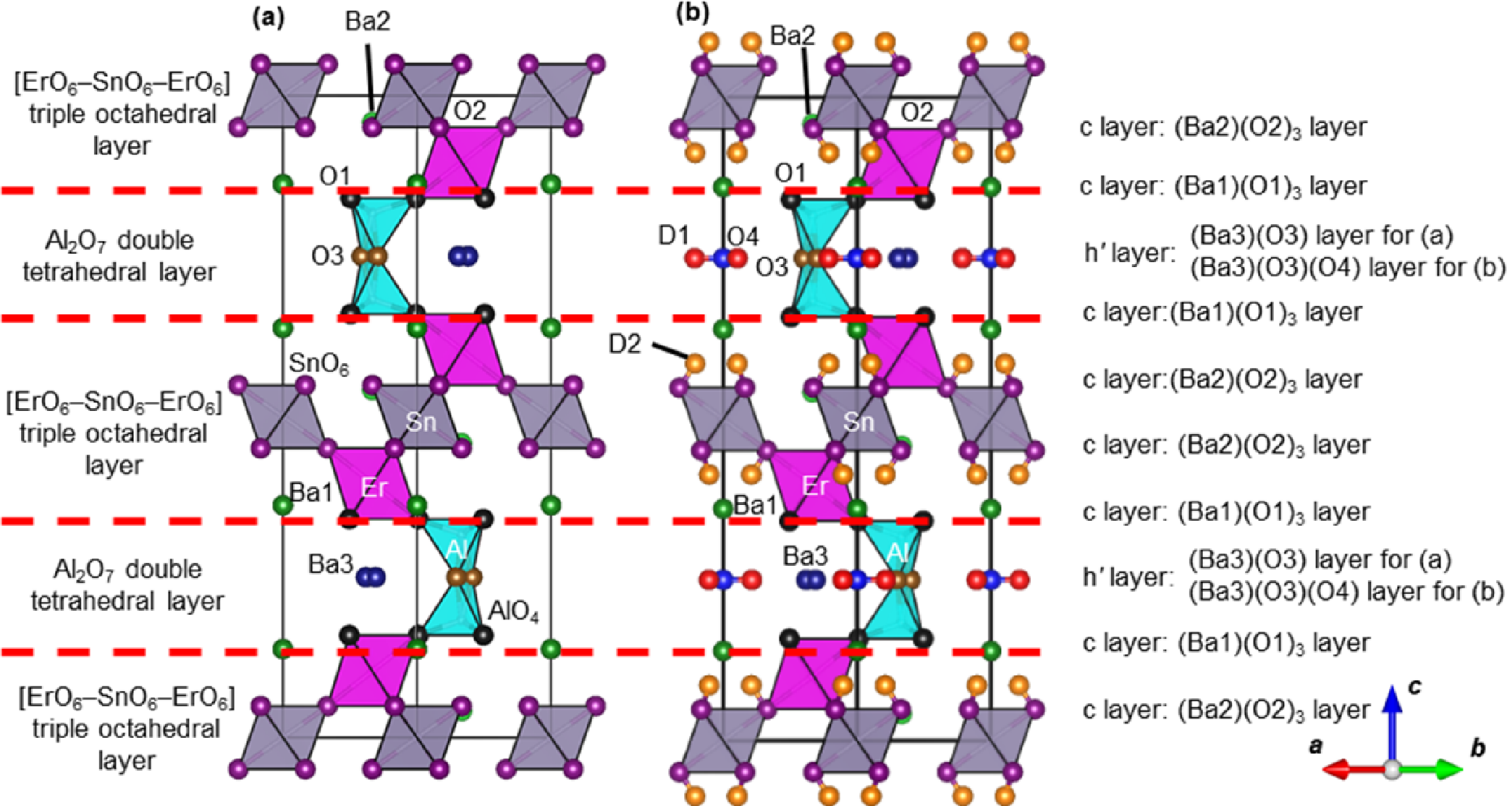
Refined crystal structures of (a) dry Ba_5_Er_2_Al_2_SnO_13_ and (b) hydrated (deuterated) Ba_5_Er_2_Al_2_SnO_12_(OD)_2_ at 5 K. Green, light-green, dark-blue, black, purple, brown, blue, red, and orange balls represent Ba1, Ba2, Ba3, O1, O2, O3, O4, D1, and D2 atoms, respectively. Pink, gray, and light-blue polyhedra represent ErO_6_ and SnO_6_ octahedra and AlO_4_ tetrahedra, respectively.

Neutron diffraction (ND) data of dry Ba_5_Er_2_Al_2_SnO_13_ and hydrated (deuterated) Ba_5_Er_2_Al_2_SnO_13–5*y*/2_(OD)_5*y*_ were measured at 5 K to investigate the bulk hydration, bulk proton concentration, and detailed crystal structure, including the occupancy and atomic coordinates of hydrogen and oxygen atoms. Rietveld refinement of the ND data of dry Ba_5_Er_2_Al_2_SnO_13_ was successfully carried out using the crystal data of hexagonal *P*6_3_/*mmc* Ba_5_Er_2_Al_2_ZrO_12.77_(OH)_0.46_ (= Ba_5_Er_2_Al_2_ZrO_13_·0.23 H_2_O) in ref (28) as initial parameters (Figure S17a and Table S3). The occupancy factor of oxygen atoms at the interstitial O4 site, *g*(O; O4), was 0.02(2), indicating no oxygen atoms at the O4 site. Thus, the O4 atom was not placed in the final refinement. The bond valence sums of all of the cations and anions for the refined structures of dry Ba_5_Er_2_Al_2_SnO_13_ were consistent with their formal charges, validating the refined crystal structure (Figure 3a).

Next, we performed Rietveld analyses of ND data of the hydrated (deuterated) Ba_5_Er_2_Al_2_SnO_13–5*y*/2_(OD)_5*y*_ pellets using the crystal data of hexagonal *P*6_3_/*mmc* Ba_5_Er_2_Al_2_ZrO_12.77_(OH)_0.46_ in ref (28) as initial parameters (Figure S17b and Table S4). The occupancy factor of oxygen atoms at the interstitial O4 site *g*(O; O4) was 1.08(3), indicating the full occupation of oxygen atoms at the O4 site. Thus, *g*(O; O4) was fixed to 1.00 in the final refinement (see the details in Table S4 and Figure S18). The bond-valence-based energy (BVE) and DFT calculations indicated the oxygen O4 and O3 sites and no other oxygen sites in the h*′* layer of BEAS (Figure S19). Therefore, the h*′* layer of fully hydrated BEAS is not the BaO_3_ layer but the BaO_2_ layer, indicating that the fully hydrated BEAS is Ba_5_Er_2_Al_2_SnO_12_(OD)_2_ (= Ba_5_Er_2_Al_2_SnO_14_D_2_). Thus, the theoretical proton concentration *y* in fully hydrated Ba_5_Er_2_Al_2_SnO_13–5*y*/2_(OH)_5*y*_ is 0.4. Rietveld analyses of hydrated BEAS were performed using 104 models with different D atom positions, which suggested two D sites of D1 closest to the O4 atom and D2 near the O2 atom. The O–D lengths obtained from the refined crystal structure of Ba_5_Er_2_Al_2_SnO_12_(OD)_2_ were 1.10 Å, which agreed with that estimated from the IR data of 0.99(4) Å (Figure S20). The lattice parameters *a* and *c* of hydrated Ba_5_Er_2_Al_2_SnO_12_(OD)_2_ were 0.34% and 0.43% higher than those of dry Ba_5_Er_2_Al_2_SnO_13_ at 5 K, respectively, due to the hydration. The bond valence sums of all of the cations and anions at all of the sites were consistent with the formal charges (Table S4). The proton concentration in Ba_5_Er_2_Al_2_SnO_13–5*y*/2_(OD)_5*y*_ calculated from the refined occupancy factors (*y* = 0.40) agreed with that from the TG data (*y* = 0.40). These results validate the refined crystal structure of Ba_5_Er_2_Al_2_SnO_12_(OD)_2_ at 5 K (Figure 3b).

Refined crystal structures indicate both dry Ba_5_Er_2_Al_2_SnO_13_ and hydrated (deuterated) Ba_5_Er_2_Al_2_SnO_12_(OD)_2_ to be 10H hexagonal perovskite-related oxides (Figure 3a,b and Supplementary Note 5). The crystal structure of dry Ba_5_Er_2_Al_2_SnO_13_ has intrinsically oxygen-deficient hexagonal close-packed Ba(O3) (h*′*) layers and triple octahedral layers (two ErO_6_ octahedral layers and one SnO_6_ octahedral layer = [ErO_6_–SnO_6_–ErO_6_] triple octahedral layer, which can be regarded as triple perovskite-like layer). On the contrary, the crystal structure of hydrated Ba_5_Er_2_Al_2_SnO_12_(OD)_2_ has BaO_2_ [= Ba(O3)(O4)] (h*′*) layers and triple octahedral layers (perovskite-like layers). In dry Ba_5_Er_2_Al_2_SnO_13_, there is no interstitial O4 atom site (*g*(O; O4) = 0.00), while in hydrated Ba_5_Er_2_Al_2_SnO_13_, the O4 site is fully occupied by oxygen atoms (*g*(O; O4) = 1.00) forming BaO_2_ (h*′*) layers. These results on *g*(O; O4) indicate that the extra oxygen atoms due to the hydration were incorporated into the interstitial oxygen O4 site and that the bulk Ba_5_Er_2_Al_2_SnO_13_ was fully hydrated under wet atmospheres (Figure S19). To the best of our knowledge, this is the first example of full hydration in hexagonal perovskite-related materials. The proton concentration calculated using the occupancy factor of the O4 atom *g*(O; O4) agreed with that calculated from TG data, indicating that the bulk proton concentration of BEAS can be determined by TG. The D atoms of hydrated Ba_5_Er_2_Al_2_SnO_12_(OD)_2_ existed at the D1 site closest to the O4 atom in the h′ Ba(O3)(O4) layers and the D2 site near the O2 atom in the octahedral layers (Figure 3b). Similarly, a fraction of the D atoms of Ba_2_LuAlO_4.522(7)_(OD)_0.957(14)_ existed in the octahedral layer.^29,34^ The presence of the D1 atom closest to the O4 atom and the D2 atom near the O2 atom observed in the refined crystal structure was consistent with the results of AIMD simulations, where protons mainly existed at the sites near the O4 and O2 atoms (Figure S21).

It is interesting to compare the crystal structure and proton conductivity of hydrated Ba_5_Er_2_Al_2_SnO_13_ with those of isostructural hydrated Ba_5_Er_2_Al_2_ZrO_13_. The lattice parameters and lattice volume of Ba_5_Er_2_Al_2_SnO_12_(OD)_2_ were smaller than those of Ba_5_Er_2_Al_2_ZrO_12.77_(OH)_0.46_ due to the smaller ionic radius of Sn^4+^ (0.69 Å for coordination number 6) compared to Zr^4+^ (0.72 Å for coordination number 6). At 200 °C in wet atmospheres, the bulk conductivity of BEAS was higher than that of Ba_5_Er_2_Al_2_ZrO_13–5*y*/2_(OH)_5*y*_ (BEAZ).^33^ The higher bulk conductivity of BEAS compared to BEAZ is attributed to the higher proton concentration of Ba_5_Er_2_Al_2_SnO_13–5*y*/2_(OH)_5*y*_ (*y* = 0.38) compared to Ba_5_Er_2_Al_2_ZrO_13–5*y*/2_(OH)_5*y*_ (*y* = 0.11) at 200 °C.^28^ At 5 K, the occupancy factor of oxygen atoms at the interstitial O4 site of hydrated Ba_5_Er_2_Al_2_SnO_12_(OD)_2_ (*g*(O; O4) = 1.00) was much higher than that of Ba_5_Er_2_Al_2_ZrO_12.77_(OH)_0.46_ (*g*(O; O4) = 0.23). The h*′* layer of hydrated BEAS (5.46(2) Å) was thicker than that of hydrated BEAZ (5.393(15) Å) (Figure S22), which corresponds to the higher occupancy at the O4 site of hydrated BEAS compared to hydrated BEAZ. It is likely that the higher proton concentration in BEAS compared to BEAZ is attributed to the higher ability to incorporate the extra oxygen atoms O4 due to hydration into the h*′* layer of BEAS compared to BEAZ.

**Figure 4:**
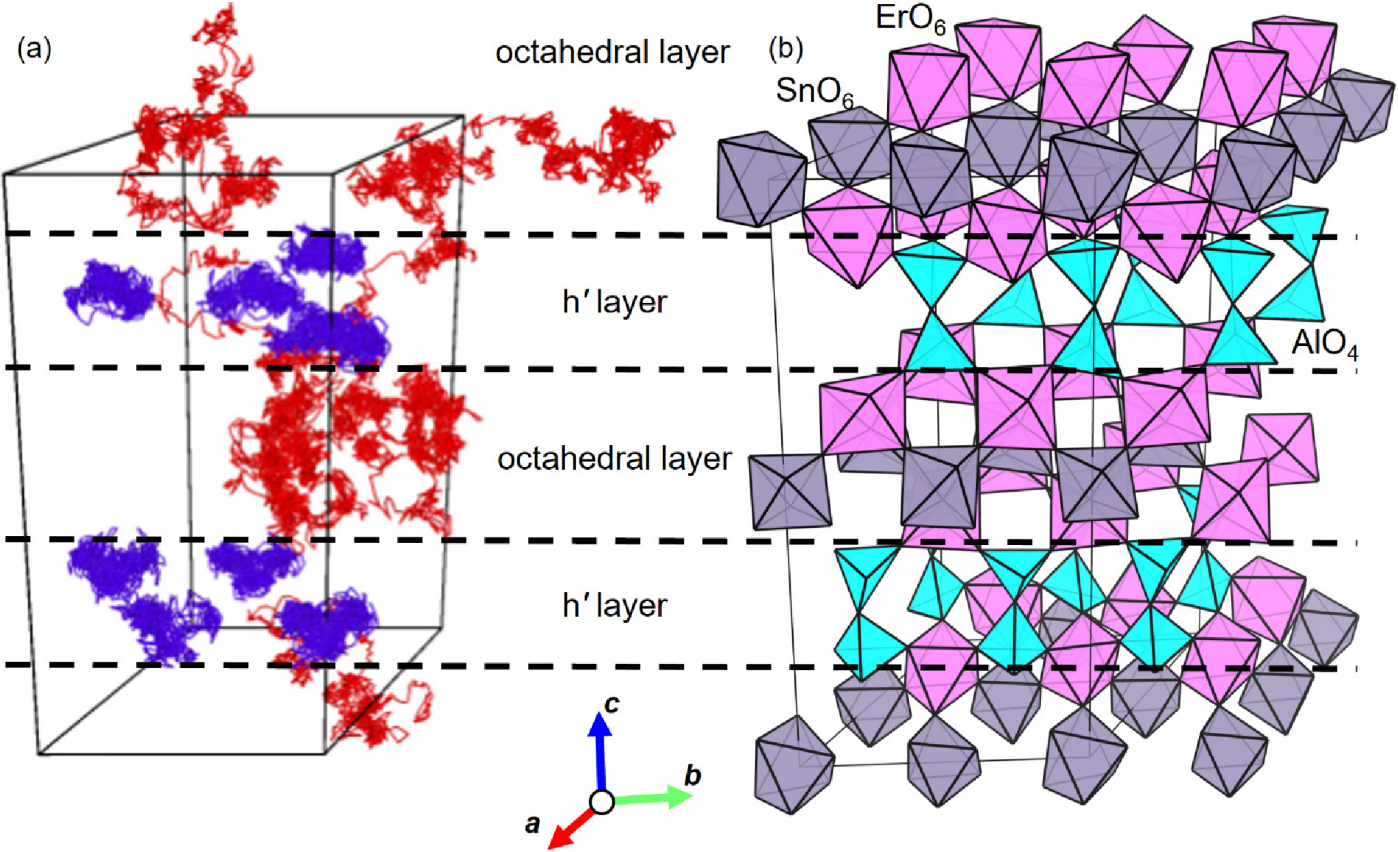
Long-range migration and trapping of protons in the octahedral and h′ layers, respectively, of BEAS, studied by the trajectories of protons from the AIMD simulations. (a) Trajectories of 16 protons of Ba_40_Er_16_Al_16_Sn_8_O_112_H_16_ in the AIMD simulations at 1200 °C during 0–10 ps. The blue lines represent the trajectories of H1–H8 placed in the h*′* layers of the initial structure. The red lines denote the trajectories of H9–H16 placed in the triple-octahedral layers of the initial structure. (b) Initial structure of Ba_40_Er_16_Al_16_Sn_8_O_112_H_16_ for AIMD simulations at 1200 °C. The pink, gray, and light-blue polyhedra stand for ErO_6_ octahedra, SnO_6_ octahedra, and AlO_4_ tetrahedra, respectively.

To investigate the proton migration in the crystal structure of BEAS, AIMD simulations of Ba_5_Er_2_Al_2_SnO_13_·H_2_O were performed at 1200 °C using a 2 × 2 × 1 supercell (Ba_40_Er_16_Al_16_Sn_8_O_112_H_16_). First, the structure of Ba_40_Er_16_Al_16_Sn_8_O_112_H_16_ was optimized by DFT calculations. The structure of Ba_40_Er_16_Al_16_Sn_8_O_112_H_16_ had two h*′* layers and two octahedral layers (Figures 4b and S23). The crystal structure refined by using the ND data of hydrated Ba_5_Er_2_Al_2_SnO_13_·D_2_O was used as the initial structure for the DFT structural optimization. Here four protons H1–H4 were placed in an h*′* layer, four protons H5–H8 were placed in the other h*′* layer, four protons H9–H12 were placed in an octahedral layer, and the other four protons H13–H16 were placed in the other octahedral layer. The optimized structure was used as the initial structure in the AIMD simulations.

**Figure 5:**
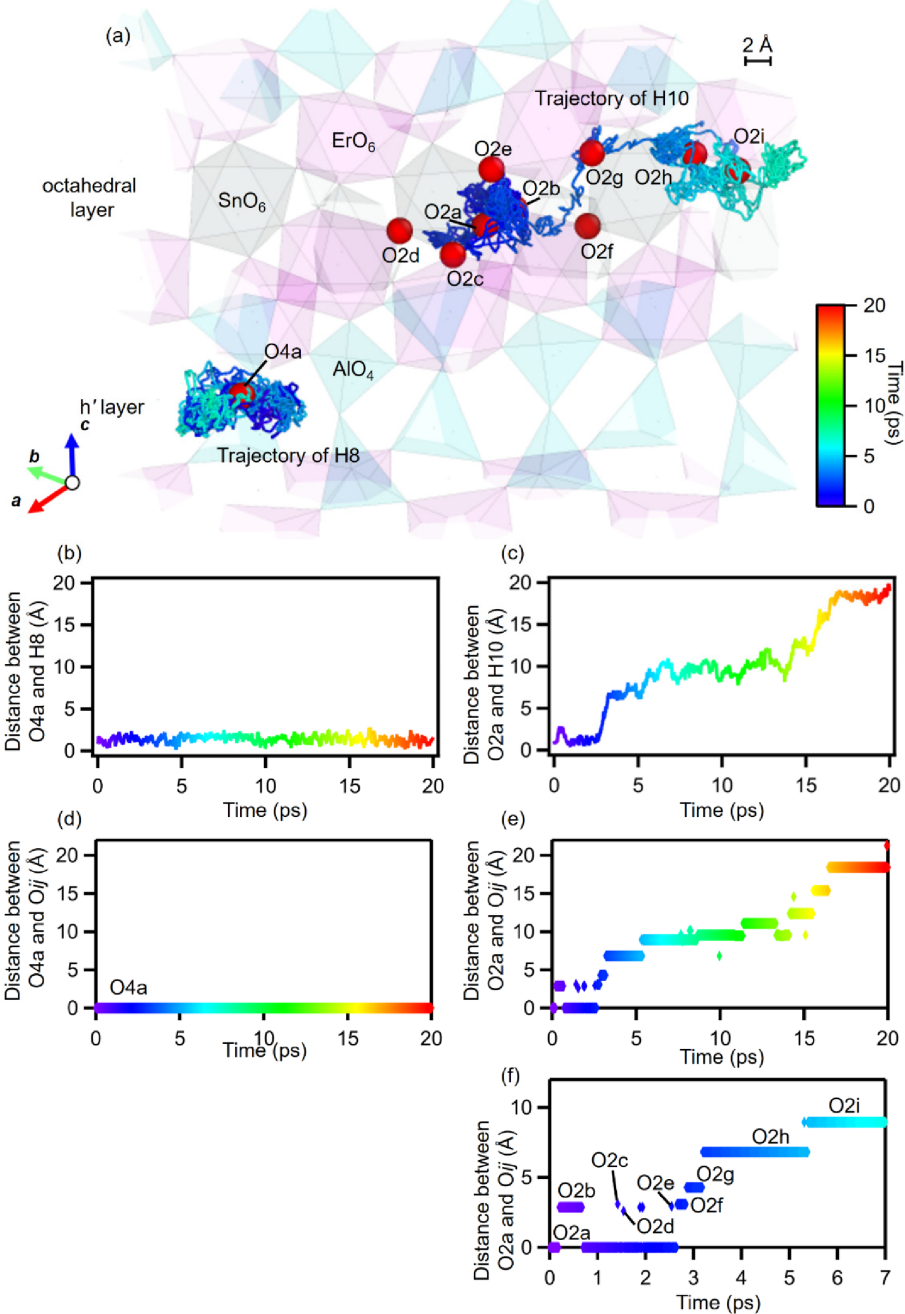
Long-range migration and trapping of protons in the octahedral and h′ layers, respectively, of BEAS, studied by the AIMD simulations of Ba_40_Er_16_Al_16_Sn_8_O_112_H_16_ at 1200 °C. (a) Rainbow trajectories of the H8 and H10 protons from 0 to 7 ps with the atomic arrangement at 0 ps. The red balls represent the oxygen atoms (O*ij*: *i* = 2, 4; *j* = a–i). The pink, gray, and light-blue polyhedra stand for ErO_6_ octahedra, SnO_6_ octahedra, and AlO_4_ tetrahedra, respectively. (b, c) Distances between (b) O4a and H8 and (c) O2a and H10 with the time evolutions from 0 to 20 ps. (d, e) Distances from (d) O4a to O*ij* and (e) O2a to O*ij* with the time evolutions, where O*ij* is closest to the H8 and H10 atom, respectively. (f) Distances between O2a and O*ij* with the time evolutions from 0 to 7 ps, where the O*ij* are shown in (a). The rainbow colors in (a–f) denote the time evolution in the AIMD simulations, as shown by the color scale in (a).

In the AIMD simulations, each proton of H1, H2, H4–H6, and H8 placed in the h*′* layer of the initial structure was mainly located closest to an O4 atom (blue trajectories in Figures 4a and S24), while each proton of H9–H13 and H15 placed in the octahedral layer of the initial structure exhibited long-range migrations (red trajectories in Figures 4a and S25) as discussed below and in Supplementary Note 6. For example, proton H8, which was placed closest to the O4a atom in the h*′* layer of the initial structure, was located closest to the O4a atom during 0–7 ps, indicating proton trapping (Figure 5a). On the contrary, proton H10, which was placed near the O2a atom in the octahedral layer of the initial structure, showed long-range migrations by reorientation and hopping from the proton sites near an O2 atom to those near a nearest-neighbor O2 atom (Figure S26 and Video S1). The H10 proton migrated between 0 and 0.2 ps from the sites near O2a to those near O2b, which we denote as “O2a–O2b”. Then the H10 proton migrated as O2b–O2a–O2c–O2a–O2d–O2a–O2e–O2a–O2f–O2g–O2h–O2i between 0.2 and 7 ps. The proton migrated from the sites near O2 to those near the nearest O2*′* along the O2–O2*′* edge of the corner-shared ErO_6_ and SnO_6_ octahedra (Figures S26 and 5a). The distance between the O4a and H8 atoms in the h*′* layer was almost constant from 0 to 20 ps (Figure 5b), which also indicates the proton trapping. In contrast, the distance between the O2a and H10 atoms increased with time, showing long-range proton diffusion (Figure 5c). For example, the distance between the O2a and H10 atoms increased from 0 Å at 0 ps to approximately 20 Å at 20 ps. The distance between the O4a atom and the O*ij* atom closest to the H8 atom was zero during 0–20 ps (Figure 5d), indicating the proton trapping (the atom labels O*ij* are shown in Figure 5a). On the contrary, the distance between O2a and the O*ij* which was nearest to H10 abruptly changed due to proton hopping to the sites near different O*ij* atoms (Figures 5e,f). The distance between the O2a and O*ij* atoms increased in a staircase-like fashion from 0 Å at 0 ps (O*ij* = O2a) to approximately 20 Å at 20 ps (O*ij* = O2i).

The MSDs of protons along the *a* and *b* directions were much higher than that along the *c* direction (e.g., 3.3 times higher at 20 ps in Figure S27), indicating that the high proton diffusion coefficient is attributable to the high proton MSDs along the *a* and *b* directions (Figure S27). The crystal structure of BEAS has the octahedral and h*′* layers, and the MSD of protons in the octahedral layers was much higher than that in the h*′* layer (e.g., 16 times higher at 20 ps in Figure S28). Therefore, the high MSDs of protons along the *a* and *b* directions are ascribed to the high MSD of protons in the octahedral layers. These results indicate that the high MSD (diffusivity) of protons in the octahedral layers is responsible for the high proton diffusion coefficient, leading to high proton conductivity.

**Figure 6:**
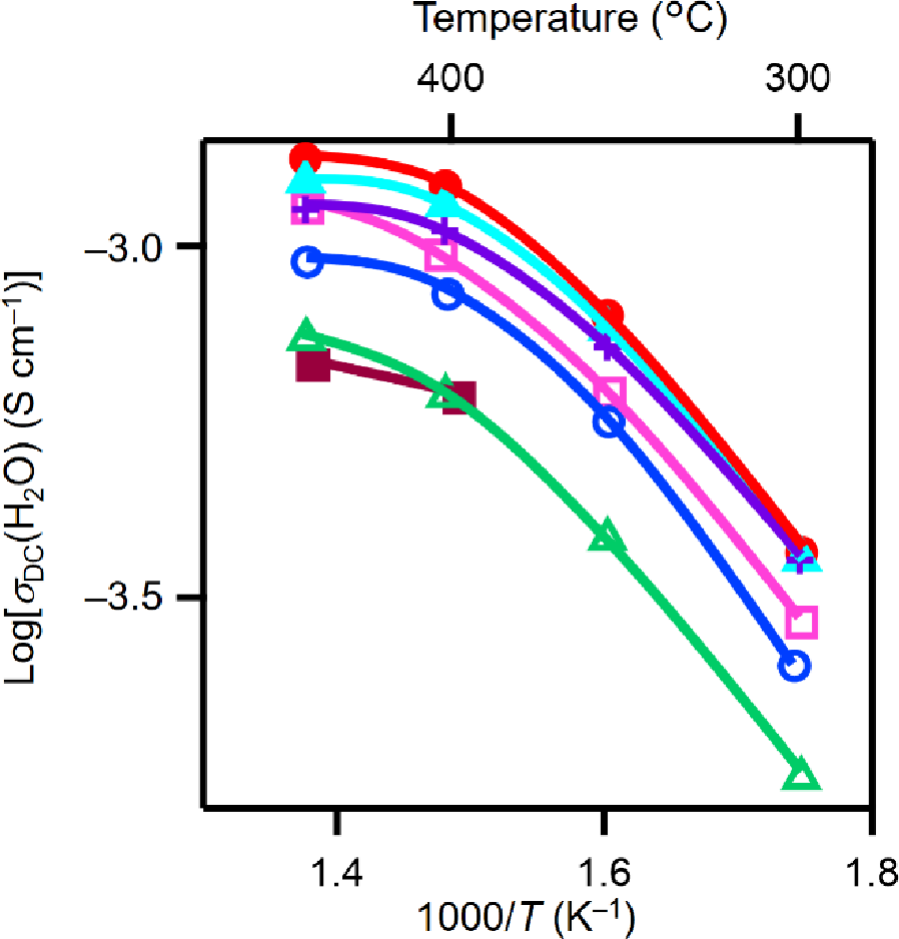
High conductivities of BRAS materials in wet air. Arrhenius plots of the DC electrical conductivity in wet air σ_DC_(H_2_O) on cooling. Red solid circles, light-blue triangles, purple + marks, pink open squares, blue open circles, green open triangles, and brown solid squares stand for the σ_DC_(H_2_O) of Ba_5_Er_2_Al_2_SnO_13–5*y*/2_(OH)_5*y*_, Ba_5_Ho_2_Al_2_SnO_13–5*y*/2_(OH)_5*y*_, Ba_5_Tm_2_Al_2_SnO_13–5*y*/2_(OH)_5*y*_, Ba_5_Dy_2_Al_2_SnO_13–5*y*/2_(OH)_5*y*_, Ba_5_Yb_2_Al_2_SnO_13–5*y*/2_(OH)_5*y*_, Ba_5_Y_2_Al_2_SnO_13–5*y*/2_(OH)_5*y*_, and Ba_5_Gd_2_Al_2_SnO_13–5*y*/2_(OH)_5*y*_, respectively.

This work has demonstrated that the new hexagonal perovskite-related oxide Ba_5_Er_2_Al_2_SnO_13–5*y*/2_(OH)_5*y*_ achieved high proton conductivity (e.g., 0.01 S cm^–1^ at 303 °C). Ba_5_*R*_2_Al_2_SnO_13–5*y*/2_(OH)_5*y*_ (*R* = rare earth) is also expected to exhibit a high proton conductivity. Therefore, we synthesized new materials Ba_5_*R*_2_Al_2_SnO_13–5*y*/2_(OH)_5*y*_ (BRAS) (*R* = Gd, Dy, Ho, Y, Tm, and Yb), as shown in Figure S29. The XRD patterns showed the formation of hexagonal perovskite-related oxides BRAS, and the lattice parameters and lattice volume increased with an increase in the ionic radius of the *R*^3+^ cation (Figure S30 and Table S5). Preliminary measurements of DC electrical conductivities were performed in wet air, which indicated high conductivities (Figure 6). These results suggest that many hexagonal perovskite-related oxides Ba_5_*R*_2_Al_2_SnO_13–5*y*/2_(OH)_5*y*_ are high proton conductors. Preliminary TG measurements of BRAS were also performed, which indicated different proton concentrations (0.19 ≤ *y* ≤ 0.40 at 100 °C) and fractional water uptakes (0.48 ≤ *F*_w_ ≤ 1.00 at 100 °C) depending on the rare earth species *R* (Figure S31). Therefore, the proton concentration, fractional water uptake, and proton conductivity were suggested to depend on not only the *M* species (*M* = Sn and Zr) but also the *R* species of the [*R*O_6_–*M*O_6_–*R*O_6_] triple octahedral layer in Ba_5_*R*_2_Al_2_*M*O_13–5*y*/2_(OH)_5*y*_.

## 3 Conclusion

In this work, we have discovered a series of new hexagonal perovskite-related oxides, Ba_5_*R*_2_Al_2_SnO_13_ (*R* = Gd, Dy, Ho, Y, Er, Tm, and Yb). Ba_5_Er_2_Al_2_SnO_13_ (BEAS) exhibits high chemical stability and high proton conductivity (e.g., 0.01 S cm^–1^ at 303 °C, which is higher compared to other proton conductors). The high proton conductivity of BEAS is attributed to its high proton concentration *y* and diffusion coefficient *D*. The higher proton conductivity of BEAS compared with the leading proton conductors BSM and BLA is attributed to the higher *D* of BEAS compared to these materials. The high *D* of BEAS is attributed to the proton diffusion in the [ErO_6_–SnO_6_–ErO_6_] octahedral layers. On the other hand, the high proton concentration in BEAS is attributed to the large amount of oxygen vacancies δ = 0.2 in Ba(Er_0.4_Sn_0.4_Al_0.2_)O_2.8−δ_ (= 0.2 Ba_5_Er_2_Al_2_SnO_13_) without water and the full hydration (high fractional water uptake *F*_w_ of 1.0). The water uptake was found to occur by the occupation of extra oxygen atoms due to hydration at the interstitial oxygen O4 site. Indeed, the neutron diffraction analyses showed that the occupancy factors of the O4 atom of dry Ba_5_Er_2_Al_2_SnO_13_ and hydrated Ba_5_Er_2_Al_2_SnO_12_(OD)_2_ are 0.00 and 1.00, respectively. Here, the occupancy factor of *g*(O4) = 1.00 also shows the full bulk hydration, as well as the TG data. To the best of our knowledge, this is the first example of full hydration in hexagonal perovskite-related materials. The fractional water uptake in the hexagonal perovskite-related oxides Ba_5_*R*_2_Al_2_*M*O_13_ (*R* = Ho, Er, and Tm for *M* = Sn; *R* = Er for *M* = Zr) is strongly dependent on the *R* and *M* species from 0.48 to 1.00 at 100 °C, resulting in the different proton concentration (*y*) values from 0.19 to 0.40 at 100 °C in Ba_5_*R*_2_Al_2_*M*O_13–5*y*/2_(OH)_5*y*_. Therefore, the fractional water uptake, proton concentration, and proton conductivity can be controlled by the chemical composition *R* and *M* species in Ba_5_*R*_2_Al_2_*M*O_13_. Previous works have focused on the *AB*O_3−δ_ perovskite-type oxides as the fast proton conductors. In this work, we have demonstrated the highest proton conductivity of novel hexagonal perovskite-related oxide Ba_5_Er_2_Al_2_SnO_13_ among the ceramic proton conductors, which would be a breakthrough for the development of fast proton conductors.

## 4 Methods

### Synthesis and Characterization of BEAS

Ba_5_Er_2_Al_2_SnO_13–5*y*/2_(OH)_5*y*_ (BEAS) samples were synthesized by solid-state reactions. The starting materials BaCO_3_ (99.95%, Kojundo Chemical Laboratory Co., Ltd., Japan), Al_2_O_3_ (99.99%, Kojundo Chemical Laboratory Co., Ltd., Japan), Er_2_O_3_ (99.9%, Shin-Etsu Chemical Co., Ltd., Japan), and SnO_2_ (99.9%, Kojundo Chemical Laboratory Co., Ltd., Japan) were mixed and ground as ethanol slurries and dry powders in an agate mortar for about 1 h. The mixture was ground with a planetary-type ball mill at a rotation speed of 300 rpm using yttria-stabilized zirconia balls in ethanol for 30 min. The mixed powders were calcined in an electric furnace at 1000 °C for 12 h in air to remove the carbonates. The calcined powders of BEAS were ground for about 1 h as dry powders, isostatically pressed into pellets at approximately 200 MPa, and sintered in air at 1500 °C for 10–20 h. We called the obtained pellets “as-prepared BEAS pellets”. The as-prepared BEAS pellets were crushed and ground in an agate mortar, and the powders thus obtained are called “as-prepared BEAS powders” and were used for Cu Kα X-ray powder diffraction (XRD), synchrotron XRD, thermogravimetric (TG), thermogravimetric–mass spectrometry (TG-MS), X-ray photoelectron spectroscopy (XPS), and IR measurements. The as-prepared BEAS pellets were used in electrical conductivity measurements and scanning electron microscopy–energy-dispersive X-ray spectroscopy (SEM-EDS) observation. The Cu Kα XRD data of the as-prepared BEAS powders were measured using RINT-2550 (Rigaku Co., Ltd., Japan) and Miniflex600 (Rigaku Co., Ltd., Japan) diffractometers with Cu Kα radiation. XPS spectra of the as-prepared BEAS powders were measured using a JPS 9010 X-ray photoelectron spectrometer (JEOL Ltd., Japan). IR data of the as-prepared BEAS powders were measured with an FT/IR-4200 (JASCO Co., Japan). The as-prepared BEAS powders were annealed at 600 °C for 12 h under wet atmospheres of O_2_, H_2_, and CO_2_ to investigate the chemical stability. The as-prepared BEAS powders were also annealed at 600 °C for 24 h under wet air to investigate the chemical stability. The SEM micrograph and EDS maps of the as-prepared BEAS pellet were acquired with a JSM-6510LA microscope (JEOL Ltd., Japan). The elemental distributions of the as-prepared BEAS pellet were homogeneous (Figure S32). TG-MS analyses of as-prepared BEAS powders were performed under a He flow at a heating rate of 20 °C min^–1^ up to 1000 °C using a Thermomass Photo system (Rigaku Co., Ltd., Japan).

### Synthesis and Characterization of Ba_5_*R*_2_Al_2_SnO_13–5*y*/2_(OH)_5_

Ba_5_*R*_2_Al_2_SnO_13–5*y*/2_(OH)_5*y*_ (BRAS) (*R* = Gd, Dy, Ho, Y, Tm, Yb, Bi, La, Nd, Sm, Eu, Lu, In, and Sc) samples were also synthesized by solid-state reactions. The starting materials BaCO_3_ (99.95%, Kojundo Chemical Laboratory Co., Ltd., Japan), Al_2_O_3_ (99.99%, Kojundo Chemical Laboratory Co., Ltd., Japan), *R*_2_O_3_ (99.9%, *R* = La, Nd, Sm, Eu, Gd, Dy, Ho, Tm, Yb, Lu and Sc, Shin-Etsu Chemical Co., Ltd., Japan; *R* = In and Bi, Kojundo Chemical Laboratory Co., Ltd., Japan; *R* = Y, Daiichi Kigenso Kagaku Kogyo Co., Ltd., Japan), and SnO_2_ (99.9%, Kojundo Chemical Laboratory Co., Ltd., Japan) were mixed and ground as ethanol slurries and dry powders in an agate mortar for about 1 h. The mixed powders were calcined in an electric furnace at 1000 °C for 12 h in air to remove carbonates. The calcined powders were ground for about 1 h as dry powders, isostatically pressed into pellets at approximately 200 MPa, and sintered in air at 1500 °C for 10–20 h. We call the pellets thus obtained as “the as-prepared BRAS pellets”. The as-prepared BRAS pellets were crushed and ground in an agate mortar into powders, which we call “the as-prepared BRAS powders”. The as-prepared BRAS powders were used for Cu Kα XRD, synchrotron XRD, and TG measurements. The Cu Kα XRD measurements of the as-prepared Ba_5_*R*_2_Al_2_SnO_13_ powders were performed using a Miniflex600 diffractometer (Rigaku Co., Ltd.) with Cu Kα radiation. We could not synthesize the hexagonal-perovskite-related oxides Ba_5_*R*_2_Al_2_SnO_13–5*y*/2_(OH)_5*y*_ (*R* = Bi, La, Nd, Sm, Eu, Lu, In, and Sc). In contrast, the hexagonal-perovskite-related oxides Ba_5_*R*_2_Al_2_SnO_13–5*y*/2_(OH)_5*y*_ (*R* = Gd, Dy, Ho, Y, Tm, and Yb) were successfully synthesized. Thus, the as-prepared BRAS pellets (*R* = Gd, Dy, Ho, Y, Tm, and Yb) were used in electrical conductivity measurements.

### Synchrotron X-ray Powder Diffraction Measurements and Data Analysis

The synchrotron XRD data of the as-prepared BRAS powders (*R* = Gd, Dy, Ho, Y, Er, Tm, and Yb) were measured in air at 27 °C in transmission geometry with six one-dimensional solid-state detectors on beamline BL02B2 at SPring-8.^48^ The wavelength was determined to be 0.4006422(7) Å using silicon powder (NIST SRM 640c). The lattice parameters of the as-prepared BRAS powders (*R* = Gd, Dy, Ho, Y, Er, Tm, and Yb) were refined by Le Bail fitting using the synchrotron XRD data and the computer program *GSAS-II*.^49^

### TG Measurements on As-Prepared Ba_5_*R*_2_Al_2_SnO_13–5*y*/2_(OH)_5*y*_

TG measurements of the as-prepared BRAS powders (*R* = Gd, Ho, Er, and Tm) were performed with a NETZSCH STA 449 F3 Jupiter system in the temperature range from 1000 to 100 °C. In the TG measurements, the as-prepared BEAS powders were heated to 1000 °C in dry N_2_ (water vapor pressure *P*(H_2_O) < 10^–4^ atm) at the heating rate of 10 °C min^–1^ and kept at 1000 °C for 1 h in dry N_2_ to dehydrate and decarbonate, and then the gas was switched to wet N_2_ (water vapor pressure *P*(H_2_O) = 0.021 atm). In the cooling process, the sample was kept for 2 h at 1000, 900, 800, 700, 600, 500, 400, 300, 200, and 100 °C to reach equilibrium in wet N_2_.

### Crystal Structure Analyses Using the Neutron Diffraction Data of Dry Ba_5_Er_2_Al_2_SnO_13_ and Hydrated Ba_5_Er_2_Al_2_SnO_12_(OD)_2_

“Dry Ba_5_Er_2_Al_2_SnO_13_” pellets were prepared by heating the as-prepared BEAS pellets at 800 °C for 30 min in a vacuum quartz tube to dehydrate and decarbonate. The sample in the quartz tube was cooled in vacuum to 300 °C, and then the quartz tube containing the pellets was sealed at this temperature. “Hydrated (deuterated) Ba_5_Er_2_Al_2_SnO_12_(OD)_2_” [“hydrated (deuterated) Ba_5_Er_2_Al_2_SnO_13–5*y*/2_(OD)_5*y*_”] pellets were synthesized using the as-prepared BEAS pellets as follows. The as-prepared BEAS pellets were heated to 1000 °C in a dry He flow and kept at this temperature in a dry He flow for 30 min to dehydrate and decarbonate, and then the dry He flow was switched to D_2_O-saturated He flow (water vapor pressure *P*(D_2_O) = 0.021 atm). The pellets were kept at 1000 °C for 2 h in the D_2_O-saturated He flow and then cooled to 100 °C at the cooling rate of 10 °C min^–1^. In the cooling process, the sample was kept in a D_2_O-saturated He flow for 2 h at each temperature of 900, 800, 700, 600, 500, 400, 300, 200, and 100 °C to reach equilibrium. Neutron diffraction data of the dry Ba_5_Er_2_Al_2_SnO_13_ and hydrated Ba_5_Er_2_Al_2_SnO_13–5*y*/2_(OD)_5*y*_ pellets were measured at 5 K with the fixed-wavelength neutron diffractometer HERMES^50^ at the JRR-3 research reactor of JAEA (Tokai, Japan) (wavelength = 1.3428(3) Å). The collected data were analyzed by the Rietveld method with the program RIETAN-FP.^51^ The bond-valence-based energy landscape for a test oxide ion in dry Ba_5_Er_2_Al_2_SnO_13_ was calculated using refined crystal parameters at 5 K with the *SoftBV* program.^52^

### Measurements of Electrical Properties

The impedance spectra of BEAS were measured in wet N_2_ (*P*(H_2_O) = 0.021 atm) and dry N_2_ (*P*(H_2_O) < 5.0 × 10^–3^ atm) using the as-prepared BEAS pellets (4 mm diameter, 6 mm thickness, relative density of 85%) with Pt electrodes. Impedance spectra were recorded with a Solartron 1260 impedance analyzer in the frequency range from 10 MHz to 0.1 Hz at an alternating voltage of 100 mV. Equivalent-circuit analysis was performed to extract the bulk conductivity and grain boundary conductivity using ZView software (Scribner Associates, Inc.). The Lin-KK software was employed to perform the Kramers–Kronig (KK) transformation on the collected impedance data.^53−55^

DC conductivities σ_DC_ of the as-prepared BEAS and BRAS pellets (*R* = Gd, Dy, Ho, Y, Tm, and Yb) were measured by a four-probe method with Pt electrodes. The temperature dependencies of σ_DC_ of the as-prepared BEAS pellets were investigated in dry and wet N_2_. The temperature dependencies of σ_DC_ of the as-prepared BEAS and BRAS pellets (*R* = Gd, Dy, Ho, Y, Tm, and Yb) were also measured in wet air. The oxygen partial pressure *P*(O_2_) dependence of σ_DC_ of the as-prepared BEAS pellets was investigated at 600, 400, and 300 °C in wet atmospheres in the *P*(O_2_) range from 1 to 2 × 10^–22^ atm. The σ_DC_ of the as-prepared BEAS pellets was also measured in dry atmospheres at 600 and 400 °C at different *P*(O_2_) in the *P*(O_2_) range from 1 to 7 × 10^–23^ atm. The *P*(O_2_) was controlled using N_2_, O_2_, and 5% H_2_ in N_2_, and monitored with an oxygen sensor at the outlet of the measurement apparatus.

### Measurements of Proton and Oxide Ion Transport Numbers

To investigate the proton transport number *t*_H_ and oxide ion transport number *t*_O_, concentration cell measurements of BEAS were performed at 500 °C by using a sintered pellet (16 mm in diameter, 1 mm thickness, and 98% relative density) attached to an alumina tube with a sealing material. The sum of *t*_H_ and *t*_O_ was estimated using the following equation based on the Nernst equation for the oxygen concentration cell:
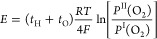
where *E*, *R*, *T*, *P*^II^(O_2_), *P*^I^(O_2_), and *F* are the electromotive force, gas constant, absolute temperature, oxygen partial pressure of the second compartment, oxygen partial pressure of the first compartment, and Faraday constant, respectively. The oxygen concentration cell is denoted as
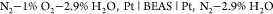
The *t*_H_ was estimated using the following equation for the water vapor concentration cell:
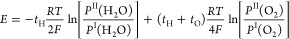
where *P*^II^(H_2_O) and *P*^II^(H_2_O) are the water vapor partial pressures of the first and second compartments, respectively. The water vapor concentration cell is denoted as
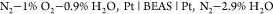


### DFT Calculations and AIMD Simulations

The static DFT calculations and AIMD simulations were performed with the Vienna Ab Initio Simulation Package (VASP)^56^ with projector augmented wave (PAW) potentials and the Perdew–Burke–Ernzerhof generalized gradient approximation (PBE-GGA). The plane-wave basis set with a cutoff of 500 eV was used in the static DFT calculations. In self-consistent cycles, the total energy was minimized until the energy convergence was less than 10^–7^ eV. Sums over occupied electronic states were calculated by the Gaussian scheme on a 7 × 7 × 2 *k*-point mesh for the 1 × 1 × 1 cell and on a 4 × 4 × 2 *k*-point mesh for the 2 × 2 × 1 cell. The cell parameters and atomic coordinates were optimized with a convergence condition of 0.02 eV Å^–1^. Possible positions of the extra interstitial oxygen atoms due to the hydration of BEAS were investigated in the h′ layer by the static DFT calculations as follows. First, the lattice parameters and atomic coordinates of the 1 × 1 × 1 cell Ba_10_Er_4_Al_4_Sn_2_O_26_ were optimized. An additional interstitial oxygen atom was then placed at the atomic coordinate (*x*, *y*, 1/4) in the optimized Ba_10_Er_4_Al_4_Sn_2_O_26_ with a step interval of 0.1 from 0 to 1 for *x* and *y* (100 positions) to calculate the total energy of Ba_10_Er_4_Al_4_Sn_2_O_27_ (Figure S19b), where the excess charge due to the interstitial oxygen atom was compensated by a uniform background charge. Structural optimization of a 2 × 2 × 1 supercell (Ba_40_Er_16_Al_16_Sn_8_O_112_H_16_) was also carried out in space group *P*1 by the DFT calculations (Figure S23). The refined crystal parameters of hydrated BEAS (Table S4) were used as the initial structure for structural optimization.

AIMD simulations were also carried out using the PAW method and the PBE-GGA for the exchange and correlation functionals. The simulations were performed on a 2 × 2 × 1 supercell (Ba_40_Er_16_Al_16_Sn_8_O_112_H_16_). The optimized structure in the static DFT calculations (Figure S23) was heated from −273 to 1200 °C at a rate of 1 °C fs^–1^. The system was further equilibrated for 2 ps, and the production trajectory was accumulated for the canonical (constant *N*, *V*, *T*) ensemble using a Nosé thermostat for ∼30 ps with a time step of 1 fs. The cutoff energy was set to 300 eV, and the reciprocal-space integration was performed only at the Γ point. To visualize the AIMD snapshots and trajectories, we used the OVITO program.^57^ The mean square displacements of all atoms were obtained with MDANSE.^58^ The refined crystal structures, the bond-valence-based energy landscape, and the probability density distribution of H atoms from the AIMD simulations were drawn with *VESTA 3*.^59^ The relative frequency of oxygens nearest to each proton (Figure S21) and time evolutions of the oxygen atoms closest to each proton (Figures S24 and S25) were obtained using the Pymatgen code.^60^

## Supporting Information Available

XRD patterns; XPS spectra; DC electrical conductivities; equivalent circuits; complex impedance plots; Arrhenius plots of bulk conductivity and grain boundary conductivity; H/D isotope effect; proton diffusion coefficient calculation; TG and TG-MS data; van’t Hoff plots; correlation among the oxygen vacancy concentration without water, proton concentration, and fractional water uptake; Rietveld patterns; refined crystal parameters and reliability factors; bond-valence-based energy landscape; total energy landscape; IR spectrum; relative frequencies of oxygens nearest to each proton; time evolutions of the oxygen atoms closest to each hydrogen atom; snapshots of the proton diffusion process; time dependence of MSDs; synchrotron XRD data; lattice parameters; and SEM micrograph and EDS maps (PDF)
Video S1: Proton diffusion process of Ba_40_Er_16_Al_16_Sn_8_O_112_H_16_ at 1200 °C from 2116 to 2878 fs (MP4)
