# Supplementary material for: High Proton Conduction in the Octahedral Layers of Fully Hydrated Hexagonal Perovskite-Related Oxides
Source: J Am Chem Soc. 2024 Jun 25;146(27):18544–55. doi: 10.1021/jacs.4c04325 (PMC11240259; doi:10.1021/jacs.4c04325)
Supplement: Supplementary file 1 — ja4c04325_si_001.pdf [file ja4c04325_si_001.pdf]

Supporting information

High proton conduction in the octahedral layers of fully hydrated hexagonal perovskite-related oxides

Kohei Matsuzaki[a], Kei Saito[a], Yoichi Ikeda[b], Yusuke Nambu[b,c,d], Masatomo Yashima[a,*]

[a] *Department of Chemistry, School of Science, Tokyo Institute of Technology, 2-12-1-W4-17, O-okayama, Meguro-ku, Tokyo 152-8551, Japan*

[b] *Institute for Materials Research, Tohoku University, 2-1-1 Katahira, Aoba-ku, Sendai 980-8577, Japan*

[c] *Organization for Advanced Studies, Tohoku University, 2-1-1 Katahira, Aoba-ku, Sendai 980-8577, Japan*

[d] *FOREST, Japan Science and Technology Agency, 4-1-8 Honcho, Kawaguchi, Saitama 332-0012, Japan*

* Corresponding author

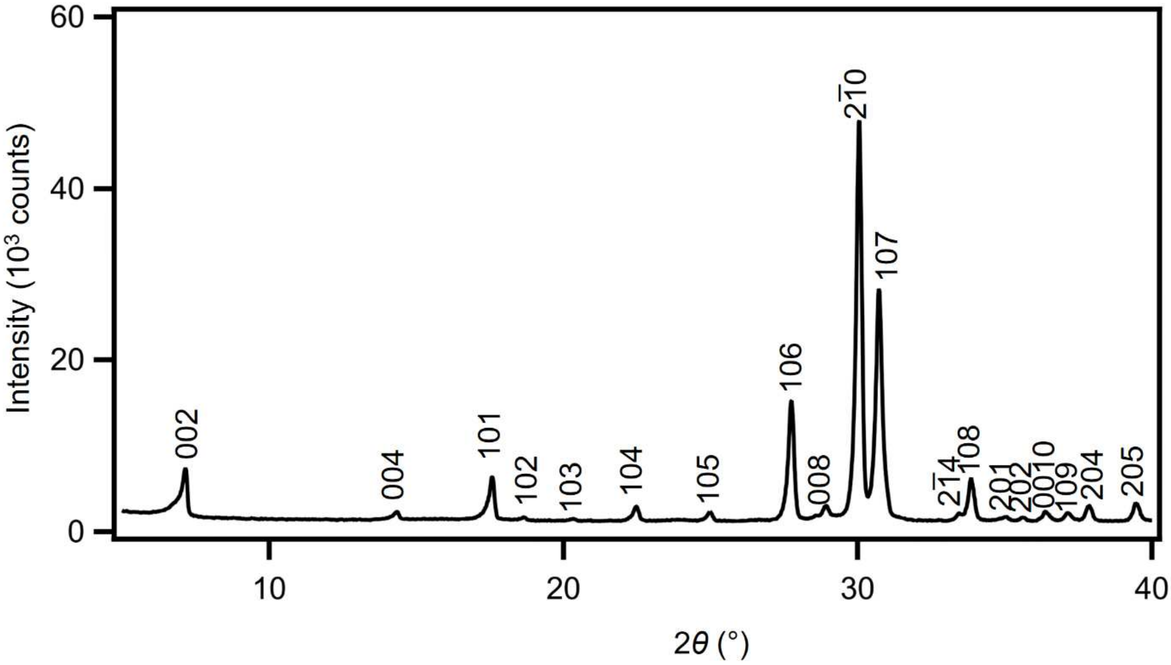

Figure S1. Cu Kα XRD pattern of the as-prepared BEAS powders. *hkl* denotes the reflection index of the primitive hexagonal lattice.

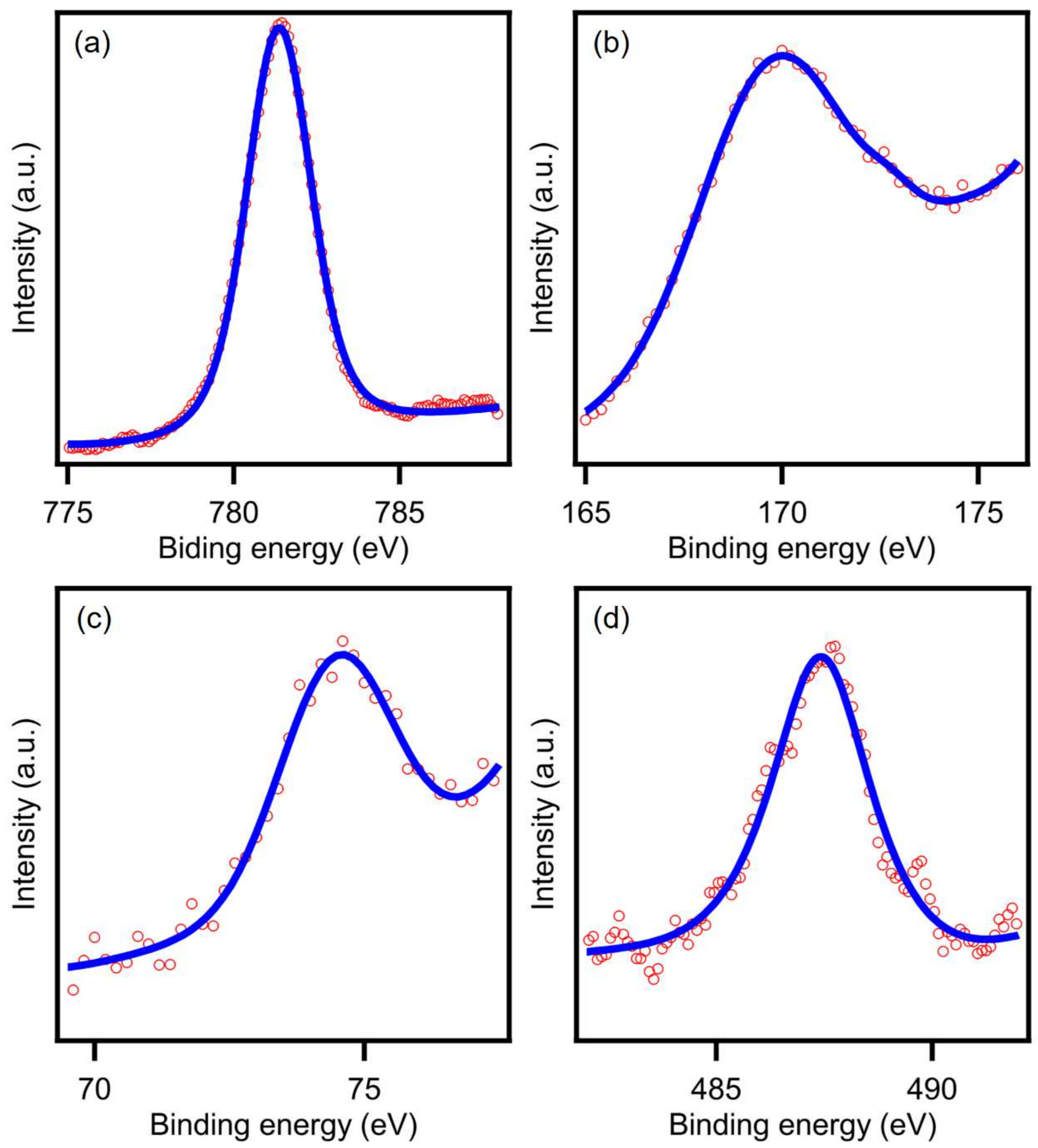

Figure S2. (a) Ba 3d, (b) Er 4d, (c) Al 2p, and (d) Sn $3d_{5/2}$ XPS spectra of the as-prepared BEAS powders. The blue line and red open circles represent the fitting line and the experimental data, respectively. The obtained binding energies of Ba 3d (780.7 eV), Er 4d (169.7 eV), Al 2p (74.5 eV), and Sn $3d_{5/2}$ (487.4 eV) indicate the oxidation states of Ba^{2+}, Er^{3+}, Al^{3+}, and Sn^{4+}, respectively.[1–4]

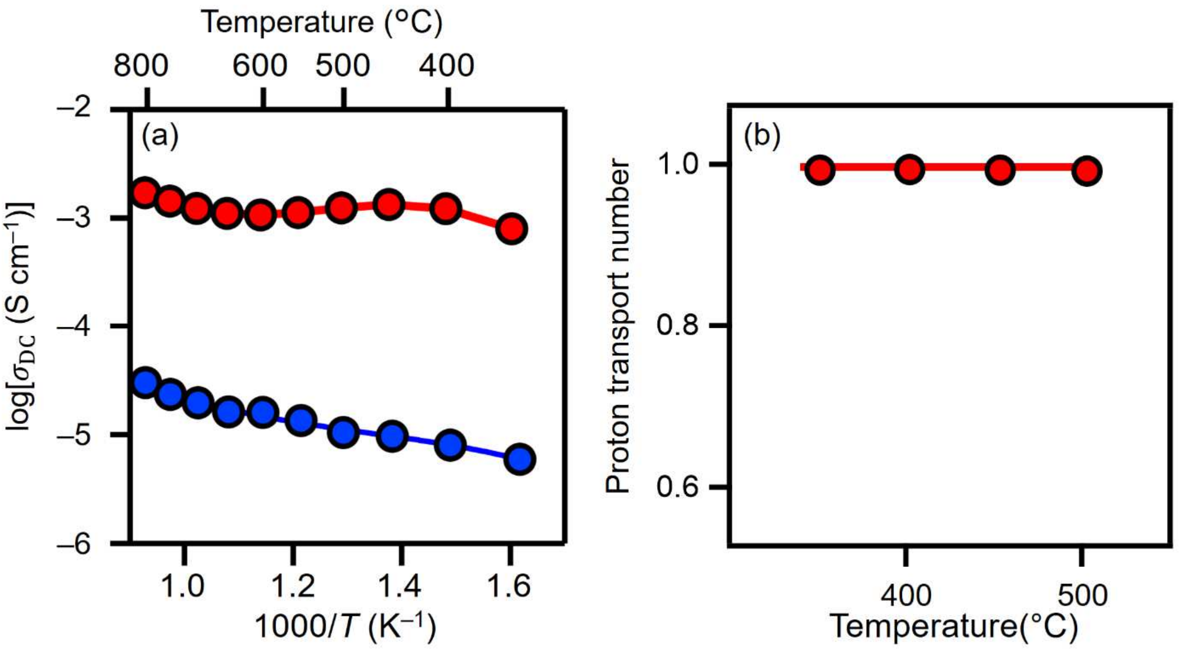

Figure S3. (a) Arrhenius plots of the DC electrical conductivity σ_{DC} of BEAS in wet N_2 ($\sigma_{DC}(H_2O)$: red solid circles and line) and dry N_2 (σ_{DC}(dry): blue solid circles and line). (b) Temperature dependence of the proton transport number $t_{DC}(H^+)$ calculated by the equation: $t_{DC}(H^+) = (\sigma_{DC}(H_2O) - \sigma_{DC}(\mathrm{dry})) / \sigma_{DC}(H_2O)$.

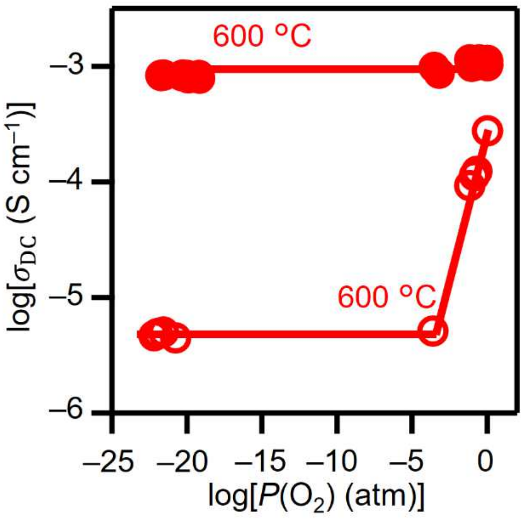

Figure S4. Oxygen partial pressure $P(O_2)$ dependencies of the DC electrical conductivity σ_{DC} of BEAS at 600 °C in wet atmospheres (red solid circles and line) and dry atmospheres (red open circles and line). The slope of log(σ_{DC}) against log($P(O_2)$) in the $P(O_2)$ range of 7×10^{-2} ~ 2×10^{-1} atm at 600 °C was 0.265(19) in dry atmospheres, which agrees with ¼ = 0.25. Therefore, the dominant charge carrier is hole in this $P(O_2)$ range.

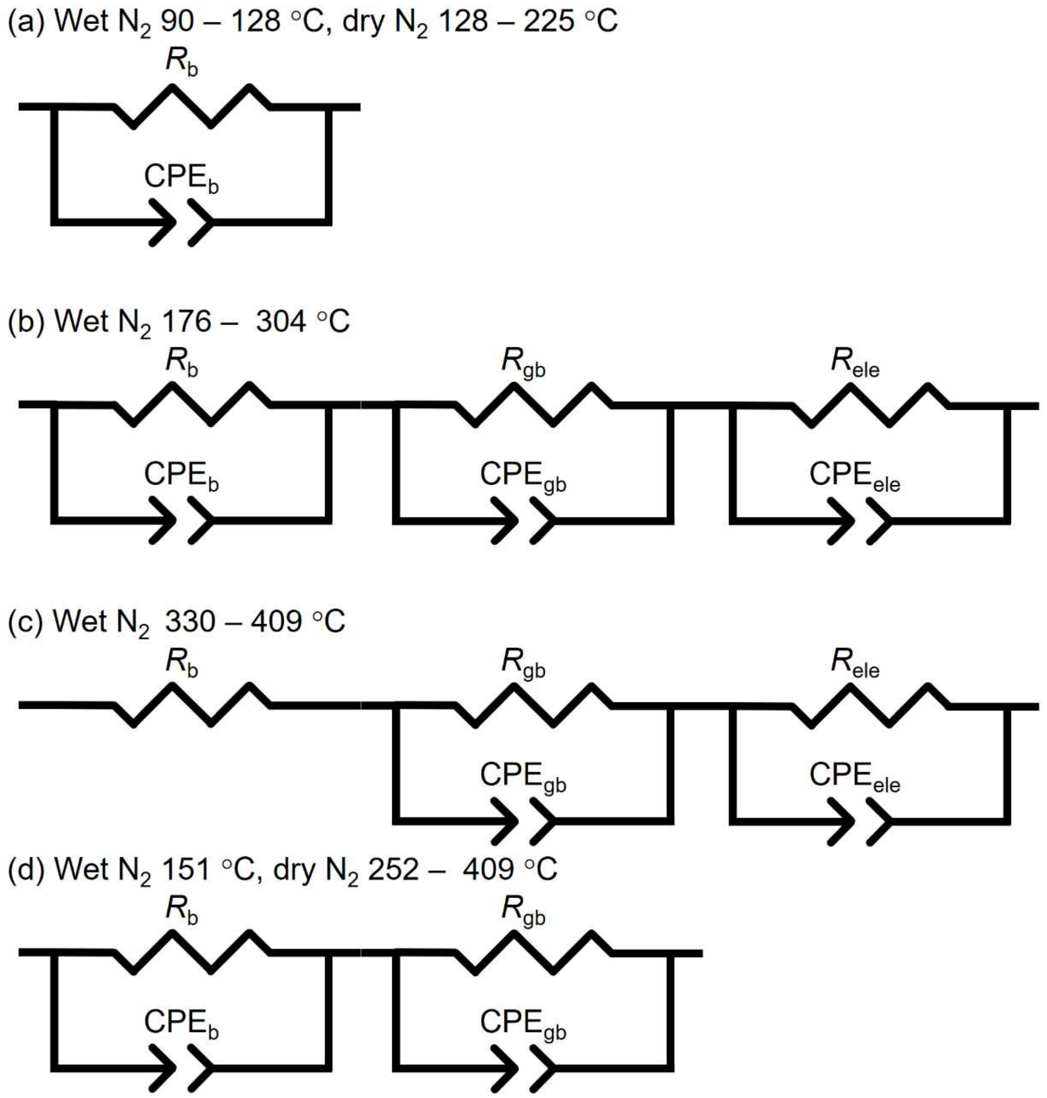

Figure S5. Equivalent circuits used to fit the impedance spectroscopy data of $Ba_5Er_2Al_2SnO_{13}{\cdot}x\,H_2O$ at (a) 128 °C, (d) 151 °C, (b) 176–304 °C, and (c) 330–409 °C in wet N_2 ($P(H_2O)$ = 0.021 atm), and $Ba_5Er_2Al_2SnO_{13}$ at (a) 128–225 °C and (d) 252–409 °C in dry N_2. *R* and CPE represent a resistor and a constant phase element, respectively. The subscripts “b”, “gb”, and “ele” stand for bulk, grain boundary, and electrode, respectively.

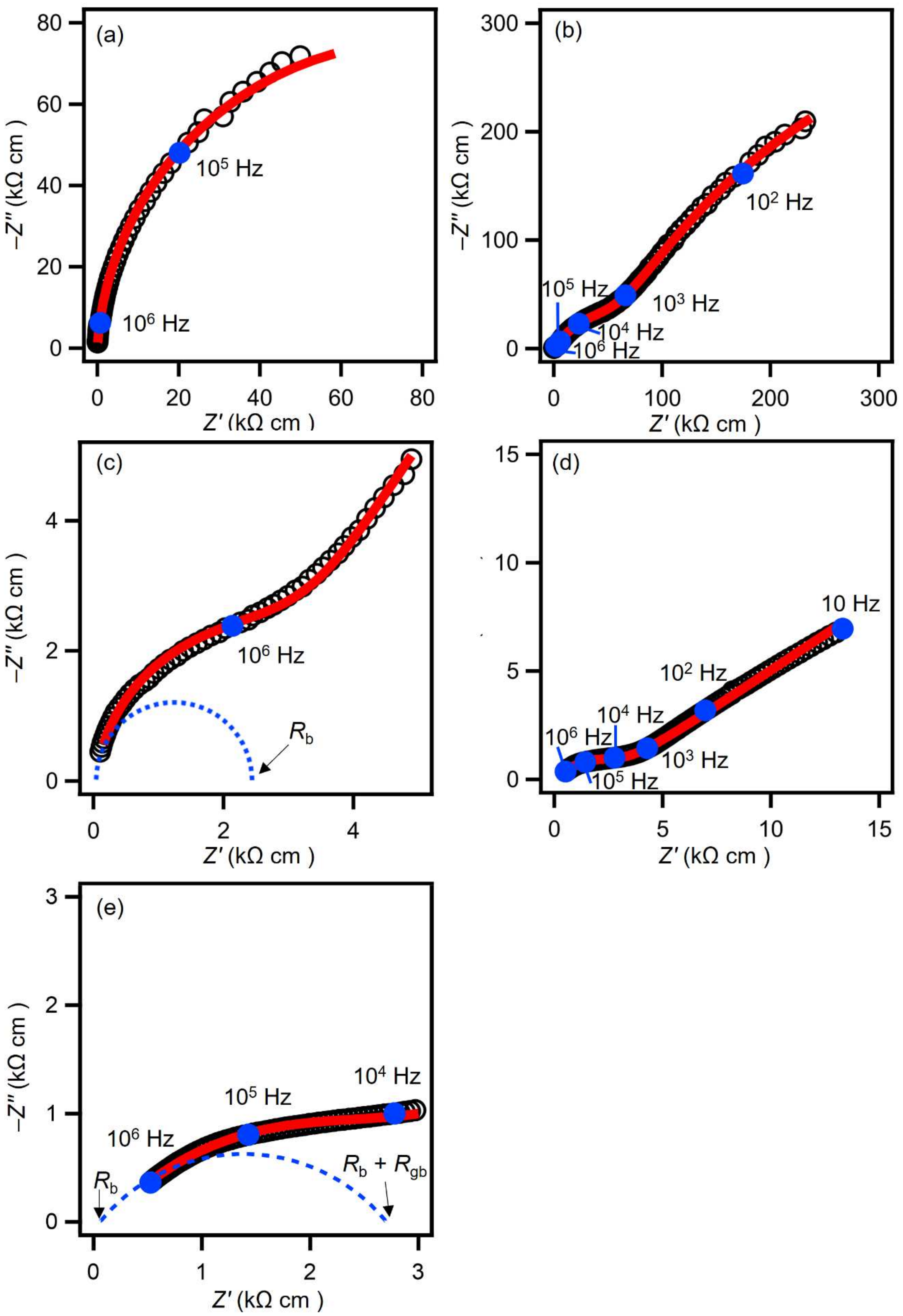

Figure S6. Complex impedance plots of BEAS at (a) 128, (b,c) 200, and (d,e) 409 °C in wet N_2 ($P(H_2O)$ = 0.021 atm). (c) and (e) are the enlarged regions showing high-frequency plots in (b) and (d), respectively. The black open circles represent the experimental data. The number of each blue solid circle denotes the frequency. The red solid line represents the calculated curve obtained by the equivalent circuit fitting (**Figures S5a, b,** and **c**).

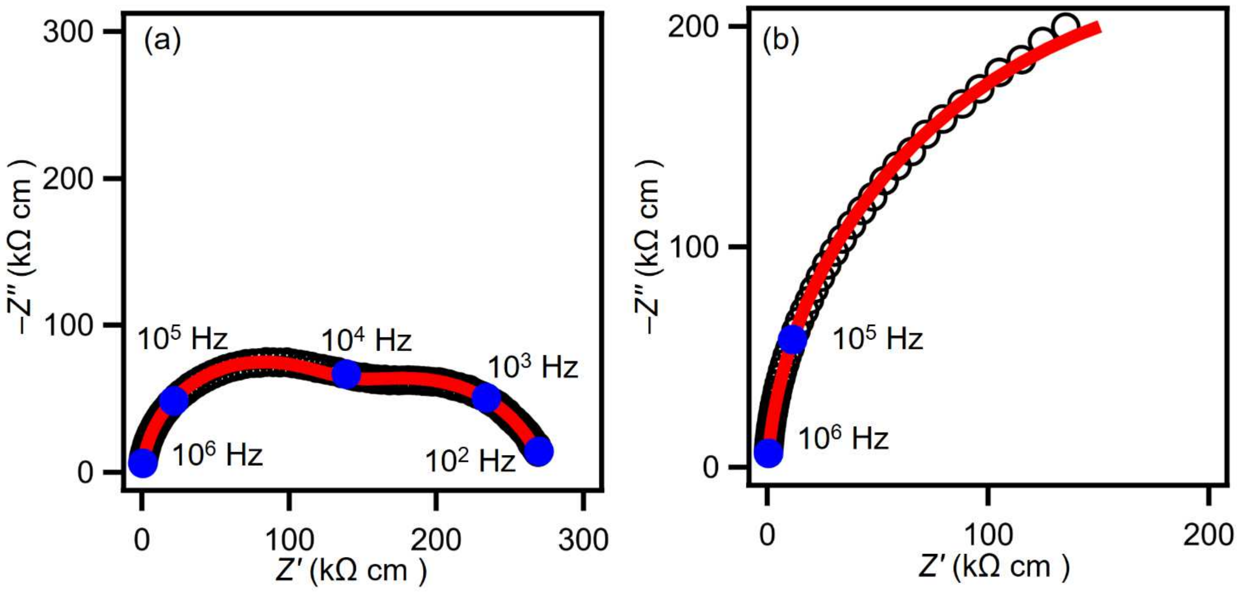

Figure S7. Complex impedance plots of BEAS at (a) 330 and (b) 225 °C in dry N_2. The number of each blue solid circle denotes the frequency. The black open circles represent the experimental data. The red solid line represents the calculated curve obtained by the equivalent circuit fitting (**Figures S5a** and **d**).

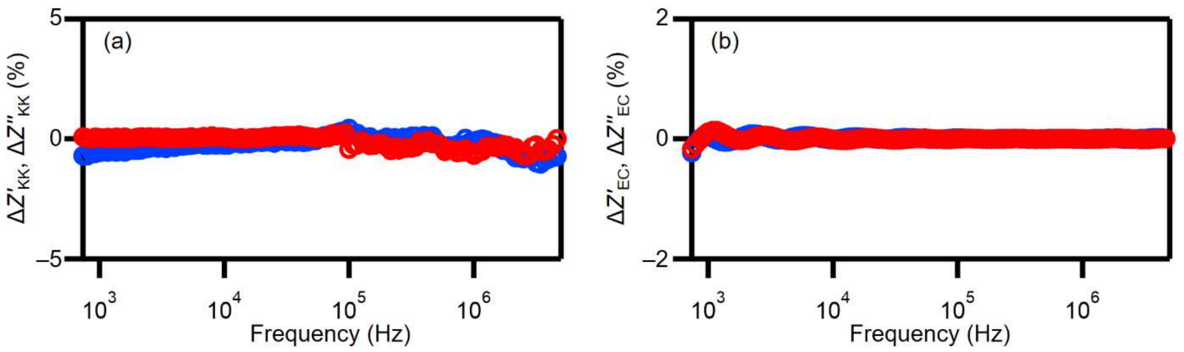

Figure S8. (a) Relative deviations between the measured impedance data and their Kramers-Kronig transform of BEAS in wet N_2 at 304 °C. (b) Residual plots for the equivalent circuit fitting of BEAS in wet N_2 at 304 °C.

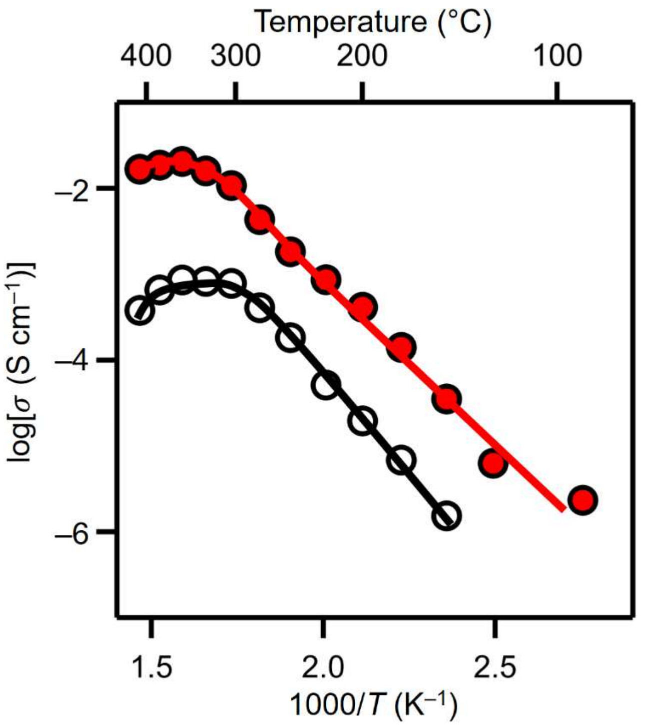

Figure S9. Arrhenius plots of bulk conductivity σ_b (red solid circles and line) and grain boundary conductivity σ_{gb} (black open circles and line) of BEAS in wet N_2. The σ_b in wet N_2 was higher than the σ_{gb} in wet N_2.

Supplementary Note 1. Higher bulk conductivity $\sigma_b(H_2O)$ of BEAS compared to other proton conductors.

Here we compare the bulk conductivity $\sigma_b(H_2O)$ of BEAS with other materials at 330 °C shown in **Figure 2a**. BEAS exhibited 1.4 times higher $\sigma_b(H_2O)$ than the leading proton conductor $BaSc_{0.8}Mo_{0.2}O_{2.8}$ (BSM) and 2.9 times higher $\sigma_b(H_2O)$ than Ba_2LuAlO_5 (BLA) at 330 °C. In addition, the $\sigma_b(H_2O)$ of BEAS was approximately 16 times higher than that of $BaZr_{0.8}Y_{0.2}O_{2.9}$ (BZY), 16 times higher than $\sigma_b(H_2O)$ of $BaCe_{0.9}Y_{0.1}O_{2.95}$ (BCY), 14 times higher than the $\sigma_b(H_2O)$ of $Ba_7Nb_4MoO_{20}$ (BNM), 5.6 times higher than the $\sigma_b(H_2O)$ of $Ba_7Nb_{3.8}Mo_{1.2}O_{20.1}$ ($BNM_{1.2}$), and 14 times higher than the $\sigma_{DC}(H_2O)$ of $Ba_5Er_2Al_2ZrO_{13}$ (BEAZ) at 330 °C.

Table S1. Bulk conductivity σ_b, grain boundary conductivity σ_{gb}, bulk capacitance C_b, grain boundary capacitance C_{gb}, and electrode capacitance C_{ele} in wet N_2.

T (°C)	σ_b (S/cm)	C_b (10^{-12} F/cm)	σ_{gb} (S/cm)	C_{gb} (10^{-11} F/cm)	C_{ele} (10^{-9} F/cm)
128	6.3×10^{-6}	0.75			
151	3.5×10^{-5}	1.1	1.5×10^{-6}	0.95	
176	1.4×10^{-4}	1.2	6.8×10^{-6}	1.4	1.6
200	4.1×10^{-4}	1.3	2.0×10^{-5}	1.6	2.0
225	8.6×10^{-4}	1.4	5.1×10^{-5}	1.7	2.8
252	1.8×10^{-3}	1.9	1.8×10^{-4}	2.3	1.8
278	4.3×10^{-3}	3.4	4.1×10^{-4}	3.2	2.5
304	1.1×10^{-2}	7.2	7.8×10^{-4}	4.1	1.2
330	1.6×10^{-2}		8.2×10^{-4}	4.4	1.2
356	2.0×10^{-2}		8.5×10^{-4}	3.7	1.9
383	1.9×10^{-2}		6.5×10^{-4}	3.2	1.9
409	1.7×10^{-2}		3.8×10^{-4}	2.8	1.2

Table S2. H/D isotope effect of BEAS on the activation energy E_a and the pre-exponential factor A for the bulk conductivities of BEAS. The E_D and A_D are the activation energy and pre-exponential factor of BEAS in D_2O-saturated N_2, respectively. E_H and A_H are the activation energy and pre-exponential factor of BEAS in H_2O-saturated N_2, respectively.

Atmosphere	$E_D - E_H$ (eV)	A_H/A_D
H_2O-saturated N_2<br>D_2O-saturated N_2	0.058(5)	0.49

The E_a and the pre-exponential factor A for the bulk conductivities were calculated using the Arrhenius equation: $\sigma T = A\exp\left(-\frac{E_a}{kT}\right)$, where σ, k, and T are the bulk conductivity, Boltzmann constant, and absolute temperature, respectively.

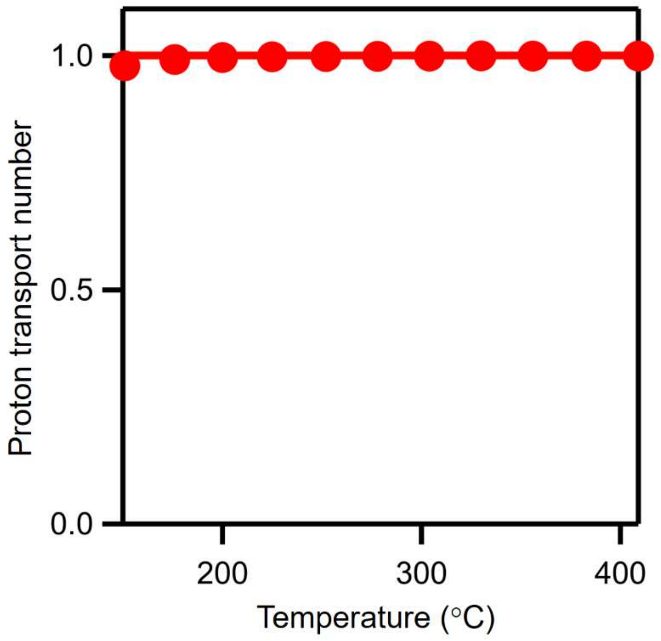

Figure S10. Temperature dependence of the proton transport number of BEAS: $\sigma_b(H^+)$ / $\sigma_b(H_2O)$.

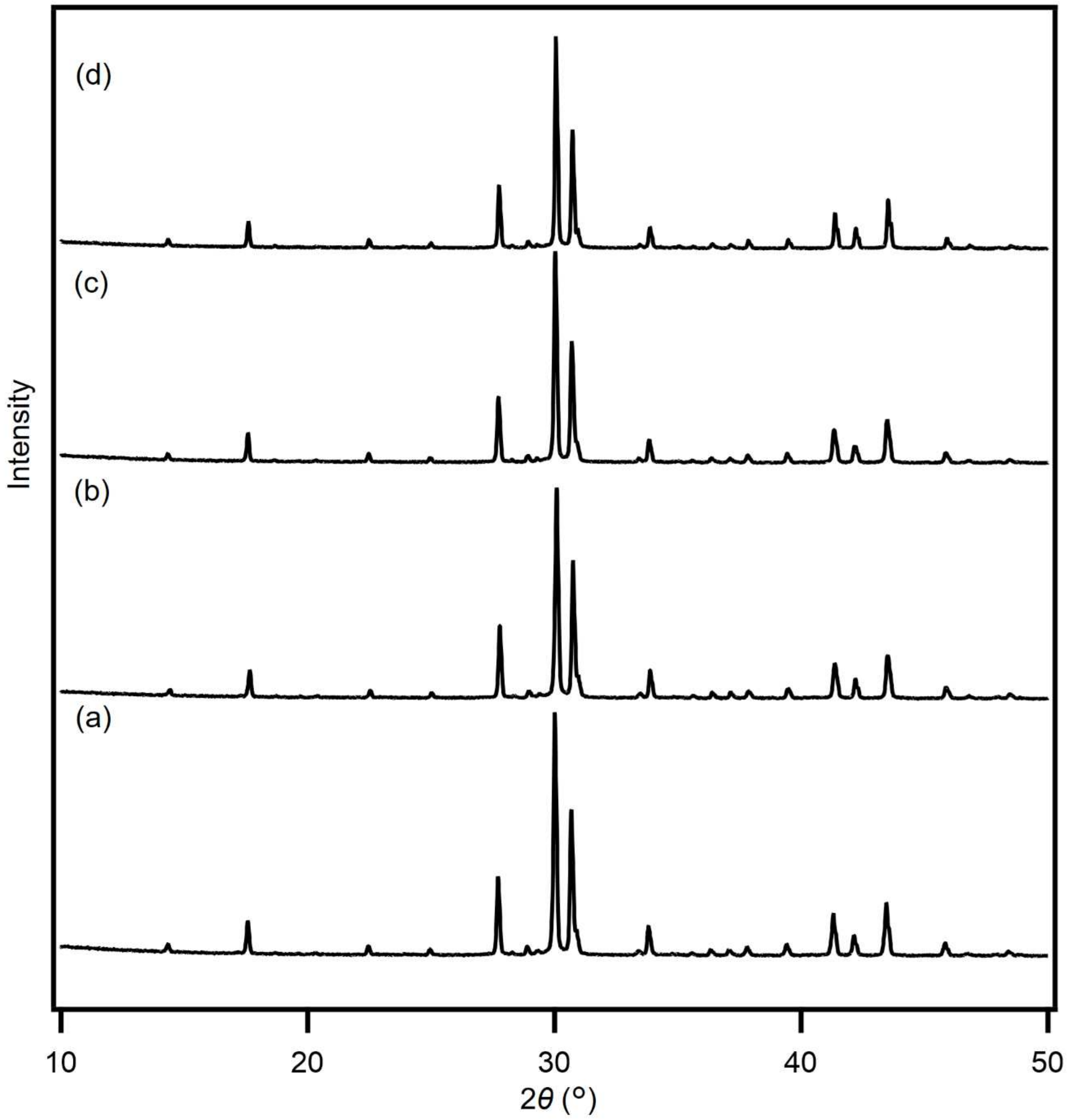

Figure S11. Cu Kα XRD patterns of (a) as-prepared BEAS powders and (b-d) BEAS powders after annealing at 600 °C for 12 h under wet atmospheres of (b) O_2, (c) H_2, and (d) CO_2 (water vapor partial pressure of 0.021 atm, 100 mL min^{-1}). There were no significant additional peaks in the XRD patterns after annealing, indicating the high chemical stability.

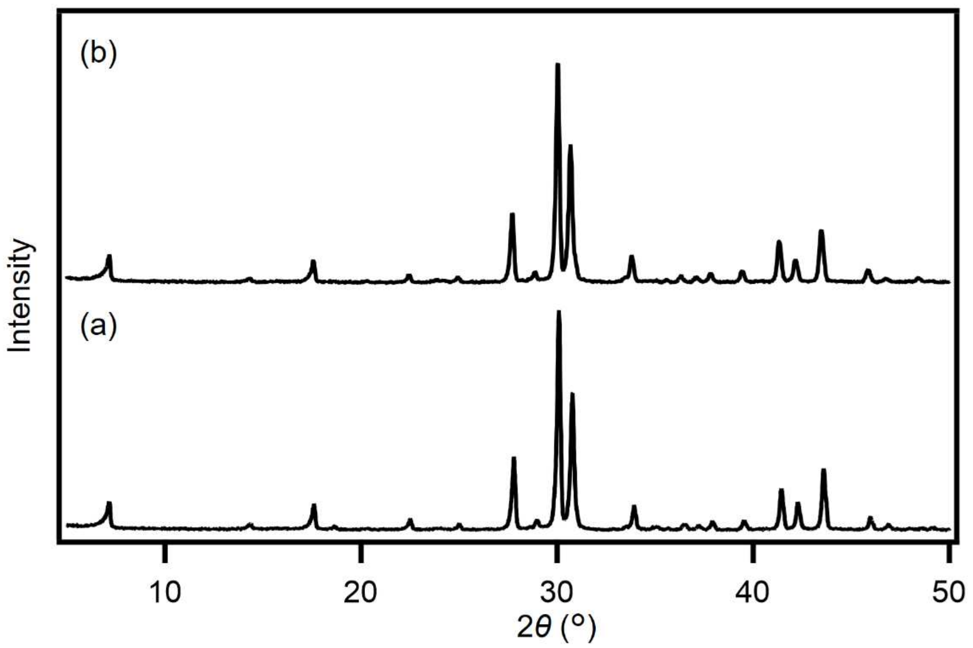

Figure S12. Cu Kα XRD patterns of (a) as-prepared BEAS powders and (b) BEAS powders after annealing at 600 °C for 24 h under wet air (water vapor partial pressure of 0.021 atm, 100 mL min^{-1}).

Supplementary Note 2. Calculation of the experimental diffusion coefficient.

The diffusion coefficient D was calculated using the Nernst-Einstein equation,

$$D = \frac{(\sigma_{\mathrm{H^+}}) \cdot RT}{F^2 C},$$

where, $\sigma_{\mathrm{H^+}}$, R, T and F are proton conductivity (**Figure 2a**), the gas constant, the absolute temperature, and the Faraday constant, respectively. C is the proton concentration estimated from TG measurements (**Figure S14**). **Figure 2b** shows the temperature dependence of D.

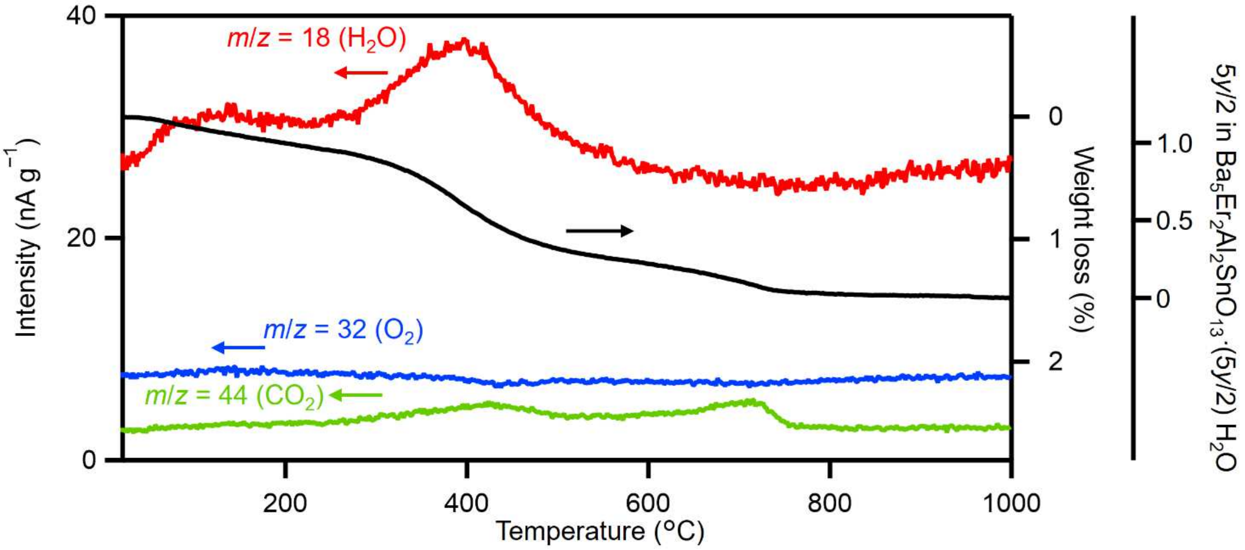

Figure S13. Thermogravimetric-mass spectrometric (TG-MS) data of as-prepared BEAS powders measured under dry He flow. The sample weight decreased during heating. MS measurements confirmed that the released gas was mainly H_2O molecules (m/z = 18, red line). Additional weight loss was observed from 300 to 800 °C due to the release of CO_2 gas (m/z = 44, green line). No significant O_2 molecules (m/z = 32, blue line) were detected.

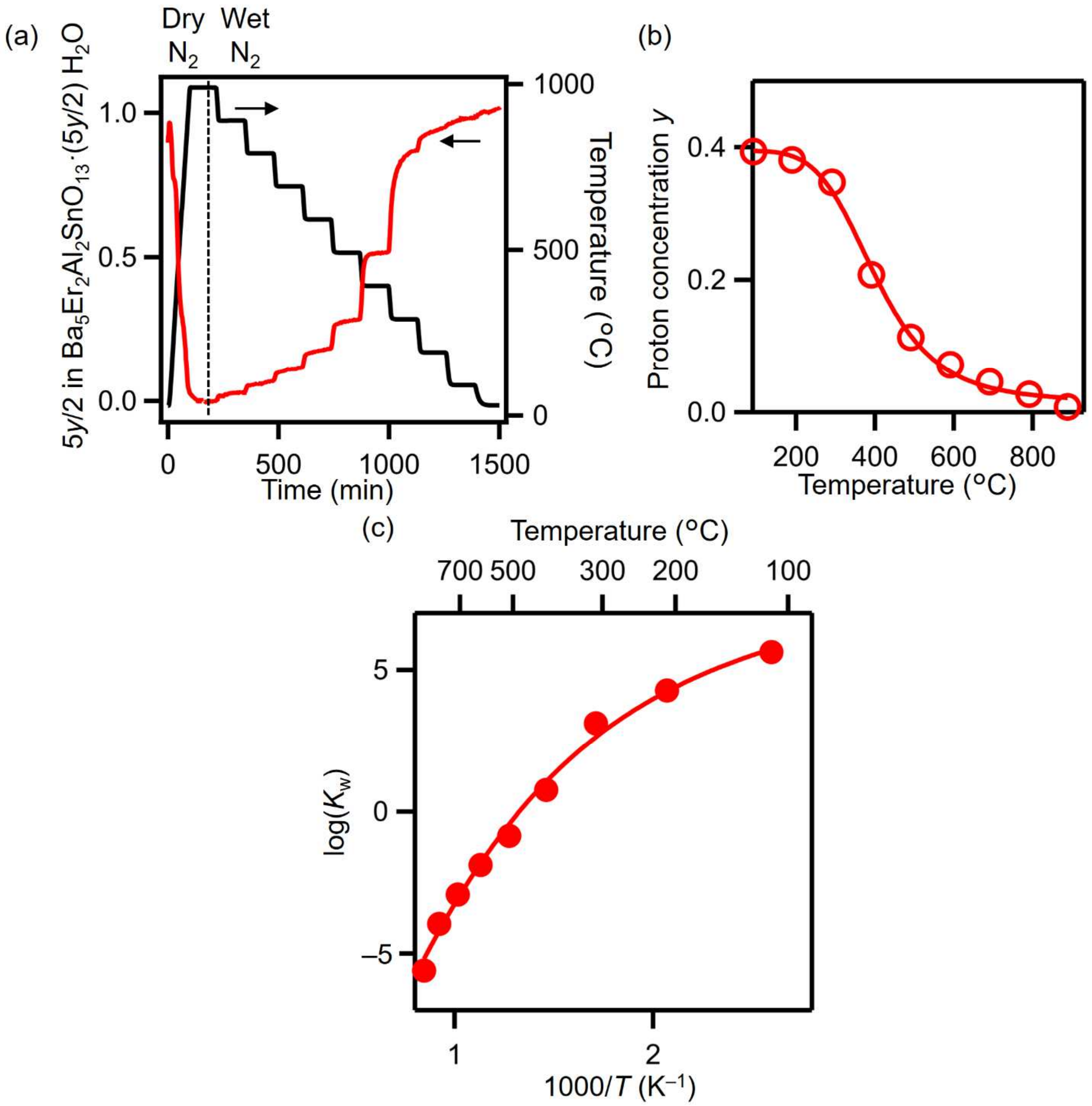

Figure S14. Water uptake of BEAS. (a) TG results of BEAS. Time dependence of temperature (black line) and the amount of water 5*y*/2 in $Ba_5Er_2Al_2SnO_{13}\cdot(5y/2)\ H_2O$ (red line) in flowing dry N_2 (time < 160 min) and wet N_2 (time > 160 min; water vapor partial pressure of 0.021 atm). The water amount (5*y*/2) in $Ba_5Er_2Al_2SnO_{13}\cdot(5y/2)\ H_2O$ was estimated by assuming that the 5*y*/2 is zero at 1000 °C in flowing dry N_2. (b) Temperature dependence of the proton concentration *y* in $Ba_5Er_2Al_2SnO_{13}\cdot(5y/2)\ H_2O$ (red open circles and line). (c) van ′t Hoff plot of the equilibrium constant K_w for the hydration reaction of $Ba_5Er_2Al_2SnO_{13}\cdot(5y/2)\ H_2O$.

Supplementary Note 3. Thermodynamics of hydration in $Ba_5Er_2Al_2SnO_{13}\cdot(5y/2)\ H_2O$ and $Ba_5Er_2Al_2ZrO_{13}\cdot(5y/2)\ H_2O$.

The hydration reaction can be described using the following Kröger-Vink notation.

$$H_2O + V_{VO}^{\times} + O_O^{\times} \rightarrow OH_{VO}' + OH_O^{\cdot}$$

Here, VO and O represent an intrinsic oxygen vacancy site O4 in the h′ layer and a lattice oxygen site (O1, O2, and O3), respectively. The hydration equilibrium constant K_w is given as

$$K_w = \frac{[(OH_{VO}')][(OH_O^{\cdot})]}{P(H_2O)[V_{VO}^{\times}][O_O^{\times}]}$$

The electroneutrality conditions, the conservations of the total number of oxygen atoms and vacancies at the interstitial O4, lattice O1, O2, and O3 sites are expressed as follows, respectively.

$$[(OH_{VO}')] = [(OH_O^{\cdot})]$$

$$[V_{VO}^{\times}] + [(OH_{VO}')] = 1$$

$$[O_O^{\times}] + [(OH_O^{\cdot})] = 13$$

As a result, the equilibrium constant K_w for the hydration can be expressed as a function of $[OH_{VO}']$.

$$K_w = \frac{[OH_{VO}']^2}{P(H_2O)(1 - [OH_{VO}'])(13 - [OH_{VO}'])} \quad (1)$$

The obtained equilibrium constants were plotted in **Figure S14c**. The hydration enthalpy (ΔH^0) and entropy (ΔS^0) of $Ba_5Er_2Al_2SnO_{13}\cdot(5y/2)\ H_2O$ were calculated using the relation of $K_w = \exp[(-\Delta H^0 + T\Delta S^0)/RT]$. The hydration enthalpies and entropies above 300 °C of $Ba_5Er_2Al_2SnO_{13}\cdot(5y/2)\ H_2O$ were calculated to be $\Delta H^0 = -78(4)\ \mathrm{kJ\ mol^{-1}}$ and $\Delta S^0 = -106(8)\ \mathrm{J\ K^{-1}\ mol^{-1}}$, which were comparable to those of other proton conductors. These values were lower than those of $Ba_5Er_2Al_2ZrO_{13}\cdot(5y/2)\ H_2O$ ($\Delta H^0 = -40(6)\ \mathrm{kJ\ mol^{-1}}$ and $\Delta S^0 = -76(9)\ \mathrm{J\ K^{-1}\ mol^{-1}}$ calculated by the equation (1) using data for proton concentration of $Ba_5Er_2Al_2ZrO_{13}\cdot x\ H_2O$ (Ref.[5]), corresponding to higher proton concentration of $Ba_5Er_2Al_2SnO_{13}\cdot(5y/2)\ H_2O$ compared to $Ba_5Er_2Al_2ZrO_{13}\cdot(5y/2)\ H_2O$.

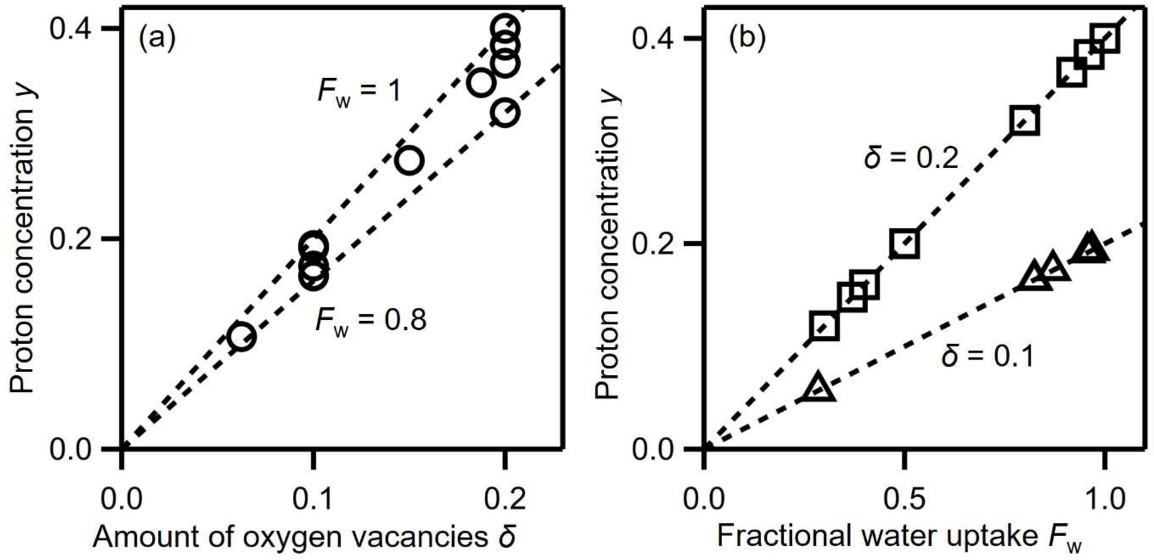

Figure S15. (a) Variation of the proton concentration y in hydrated perovskite and hydrated hexagonal perovskite-related oxides with the amount of oxygen vacancies δ in dry materials (open circles). Black dashed lines represent the calculated proton concentration y for the fractional water uptake F_w values of 0.8 and 1 in hydrated materials. (b) Variation of the proton concentration y in hydrated perovskite and hydrated hexagonal perovskite-related oxides with the fractional water uptake F_w. Black squares and triangles denote the materials with $\delta = 0.1$ and 0.2, respectively. Black dashed lines represent the calculated proton concentration y for $\delta = 0.1$ and 0.2 in hydrated materials.

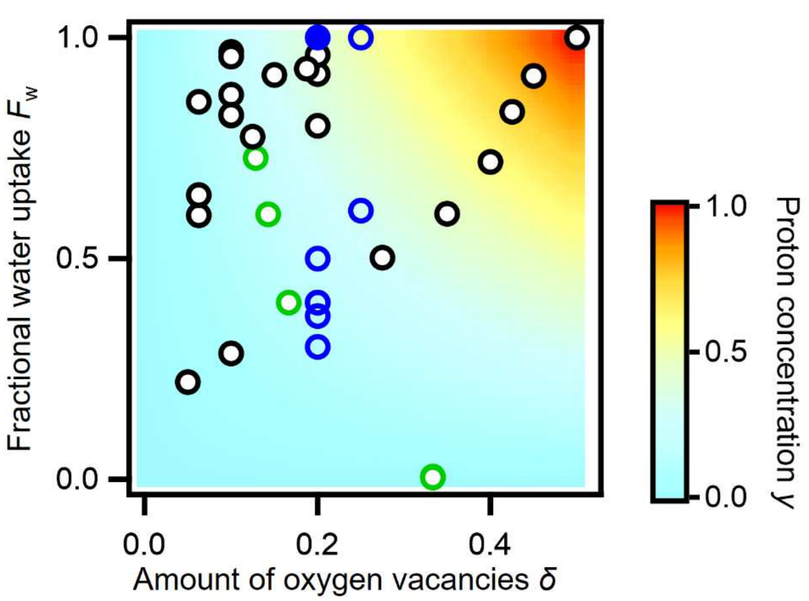

Figure S16. Plots of the fractional water uptake F_w (= $y/2\delta$) in hydrated perovskite-type and hexagonal perovskite-related oxides against the amount of oxygen vacancies δ in dry materials, showing the proton concentration y of hydrated materials. Blue solid circle stands for the data of BEAS, and black, green, blue open circles denote the data of $BaBO_{3-\delta}\cdot(y/2)\ H_2O$ perovskites and perovskite-related materials, B-site deficient hexagonal perovskite-related materials with the intrinsically oxygen deficient cubic close-packed c′ layer $BaB_{1-x}O_{3-\delta}\cdot(y/2)\ H_2O$ (x=1/3, 2/7), and $BaBO_{2.8-\delta}\cdot(y/2)\ H_2O$ hexagonal perovskite-related materials with h′ layer, respectively. Here, B cations are relatively smaller cations than Ba^{2+}.

Supplementary Note 4.

(1) Larger amount of oxygen vacancies and higher fractional water uptake of BEAS compared to other proton conductors.

The higher proton concentration y = 0.4 of BEAS (= $Ba_5Er_2Al_2SnO_{14-5\delta}\cdot(5y/2)\ H_2O$ = $Ba_5(Er_{2/5}Al_{2/5}Sn_{1/5})_5O_{(14/5-\delta)5}\cdot(5y/2)\ H_2O$ = 5 $Ba(Er_{0.4}Al_{0.4}Sn_{0.2})O_{2.8-\delta}\cdot(y/2)\ H_2O$) is attributed to the larger amount of oxygen vacancies δ = 0.2 of BEAS without water compared to $Ba_7Nb_{3.8}Mo_{1.2}O_{21-7\delta}\cdot(7y/2)\ H_2O$ (=$Ba_7(Nb_{3.8/7}Mo_{1.2/7})_7O_{(21-7\delta)/7}\cdot(7y/2)\ H_2O$ = 7 $Ba(Nb_{3.8/7}Mo_{1.2/7})O_{3-\delta}\cdot(y/2)\ H_2O$) ($\delta$ = 0.13) without water and $BaZr_{0.8}Y_{0.2}O_{3-\delta}\cdot(y/2)\ H_2O$ (δ = 0.1) and $BaCe_{0.9}Y_{0.1}O_{3-\delta}\cdot(y/2)\ H_2O$ (δ= 0.05) (Ref.[6]), and higher fractional water uptake F_w = 1.0 of BEAS compared to $Ba_5Er_2Al_2ZrO_{14-5\delta}\cdot(5y/2)\ H_2O$ (= $Ba_5(Er_{2/5}Al_{2/5}Zr_{1/5})_5O_{(14/5-5\delta)5}\cdot(5y/2)\ H_2O$ = 5 $Ba(Er_{0.4}Al_{0.4}Zr_{0.2})O_{2.8-\delta}\cdot(y/2)\ H_2O$) ($F_w$ = 0.23) (Ref.[5]), $Ba_3NbMoO_{9-3\delta}\cdot(3y/2)\ H_2O$ (= $Ba_3(Nb_{1/3}Mo_{1/3})_3O_{(3-\delta)3}\cdot(3y/2)\ H_2O$ = $Ba(Nb_{1/3}Mo_{1/3})O_{(3-\delta)}\cdot(y/2)\ H_2O$) ($F_w$ = 0.4) (Ref.[7]), and $Ba(Sc_{0.8}Mo_{0.2})O_{3-\delta}\cdot(y/2)\ H_2O$ (F_w = 0.8) (Ref.[8]).

(2) High proton concentration y of other materials (**Figure S16**)

The y of BEAS (y = 0.4) is lower than that of $Ba_2LuAlO_{5.5-2\delta}\cdot y\ H_2O$ (= $Ba_2(Lu_{0.5}Al_{0.5})_2O_{(5.5/2-\delta)2}\cdot y\ H_2O$ = $Ba(Lu_{0.5}Al_{0.5})O_{2.75-\delta}\cdot y/2\ H_2O$ = $Ba(Lu_{0.5}Al_{0.5})O_{2.5-y/2}(OH)_y$; y = 0.5) (Ref.[9]), because the amount of oxygen vacancies δ multiplied by the fractional water uptake F_w of BEAS (δF_w) is lower than (δF_w) of $Ba_2LuAlO_{5.5-2\delta}\cdot y\ H_2O$. The y of BEAS (y = 0.4) is also lower than that of $Ba_2In_2O_{6-2\delta}\cdot y\ H_2O$ (= $Ba_2In_2O_{(3-\delta)2}\cdot y\ H_2O$ = $BaInO_{3-\delta}\cdot y/2\ H_2O$ = $BaInO_{2.5-y/2}(OH)_y$; y = 1) (Ref.[10]), because the amount of oxygen vacancies δ multiplied by the fractional water uptake F_w of BEAS (δF_w = 0.2 × 1 = 0.2) is lower than of $Ba_2In_2O_{5-2\delta}\cdot y\ H_2O$ (δF_w = 0.5 × 1 = 0.5). The y of BEAS (y = 0.4) is lower than that of $Ba_2In_{1.9}P_{0.1}O_{6-2\delta}\cdot y\ H_2O$ (= $Ba_2(In_{0.95}P_{0.05})_2O_{(3-\delta)2}\cdot y\ H_2O$ = $BaIn_{0.95}P_{0.05}O_{3-\delta}\cdot y/2\ H_2O$ = $BaIn_{0.95}P_{0.05}O_{2.55-y/2}(OH)_y$; y = 0.82) (Ref.[10]), because the amount of oxygen vacancies δ multiplied by the fractional water uptake F_w of BEAS (δF_w =0.2 × 1 =0.2) is lower than of $Ba_2In_{1.9}P_{0.1}O_{6-2\delta}\cdot y\ H_2O$ (δF_w = 0.45 × 0.91 = 0.41). The y of BEAS (y = 0.4) is lower than that of $Ba_2In_{1.9}S_{0.1}O_{6-2\delta}\cdot y\ H_2O$ (= $Ba_2(In_{0.95}S_{0.05})_2O_{(3-\delta)2}\cdot y\ H_2O$ = $BaIn_{0.95}S_{0.05}O_{3-\delta}\cdot y/2\ H_2O$ = $BaIn_{0.95}S_{0.05}O_{2.575-y/2}(OH)_y$; y = 0.70) (Ref.[10]), because the amount of oxygen vacancies δ multiplied by the fractional water uptake F_w of BEAS (δF_w =0.2 × 1 = 0.2) is lower than of $Ba_2In_{1.9}S_{0.1}O_{6-2\delta}\cdot y\ H_2O$ (δF_w = 0.425 × 0.83 = 0.35). The y of BEAS (y = 0.4) is lower than that of $Ba_2In_{1.8}P_{0.2}O_{6-2\delta}\cdot y\ H_2O$ (= $Ba_2(In_{0.9}P_{0.1})_2O_{(3-\delta)2}\cdot y\ H_2O$ = $Ba(In_{0.9}P_{0.1})O_{3-\delta}\cdot y/2\ H_2O$ = $Ba(In_{0.9}P_{0.1})O_{2.6-y/2}(OH)_y$; y = 0.57) (Ref.[10]), because the amount of oxygen vacancies δ multiplied by the fractional water uptake F_w of BEAS (δF_w =0.2 × 1 =0.2) is lower than of $Ba_2In_{1.8}P_{0.2}O_{6-2\delta}\cdot y\ H_2O$ (δF_w = 0.4 × 0.72 = 0.29). The y of BEAS (y = 0.4) is lower than that of $Ba_2In_{1.8}S_{0.2}O_{6-2\delta}\cdot y\ H_2O$ (= $Ba_2(In_{0.9}S_{0.1})_2O_{(3-\delta)2}\cdot y\ H_2O$ = $Ba(In_{0.9}S_{0.1})O_{3-\delta}\cdot y/2\ H_2O$ = $Ba(In_{0.9}S_{0.1})O_{2.65-y/2}(OH)_y$; y = 0.42) (Ref.[10]), because the amount of oxygen vacancies δ multiplied by the fractional water uptake F_w of BEAS (δF_w = 0.2 × 1 = 0.2) is lower than of $Ba_2In_{1.8}S_{0.2}O_{6-2\delta}\cdot y\ H_2O$ (δF_w = 0.35 × 0.60 = 0.21). However, the bulk conductivity of BEAS is higher than those of these materials, due to higher proton diffusion coefficient of BEAS compared to these materials.

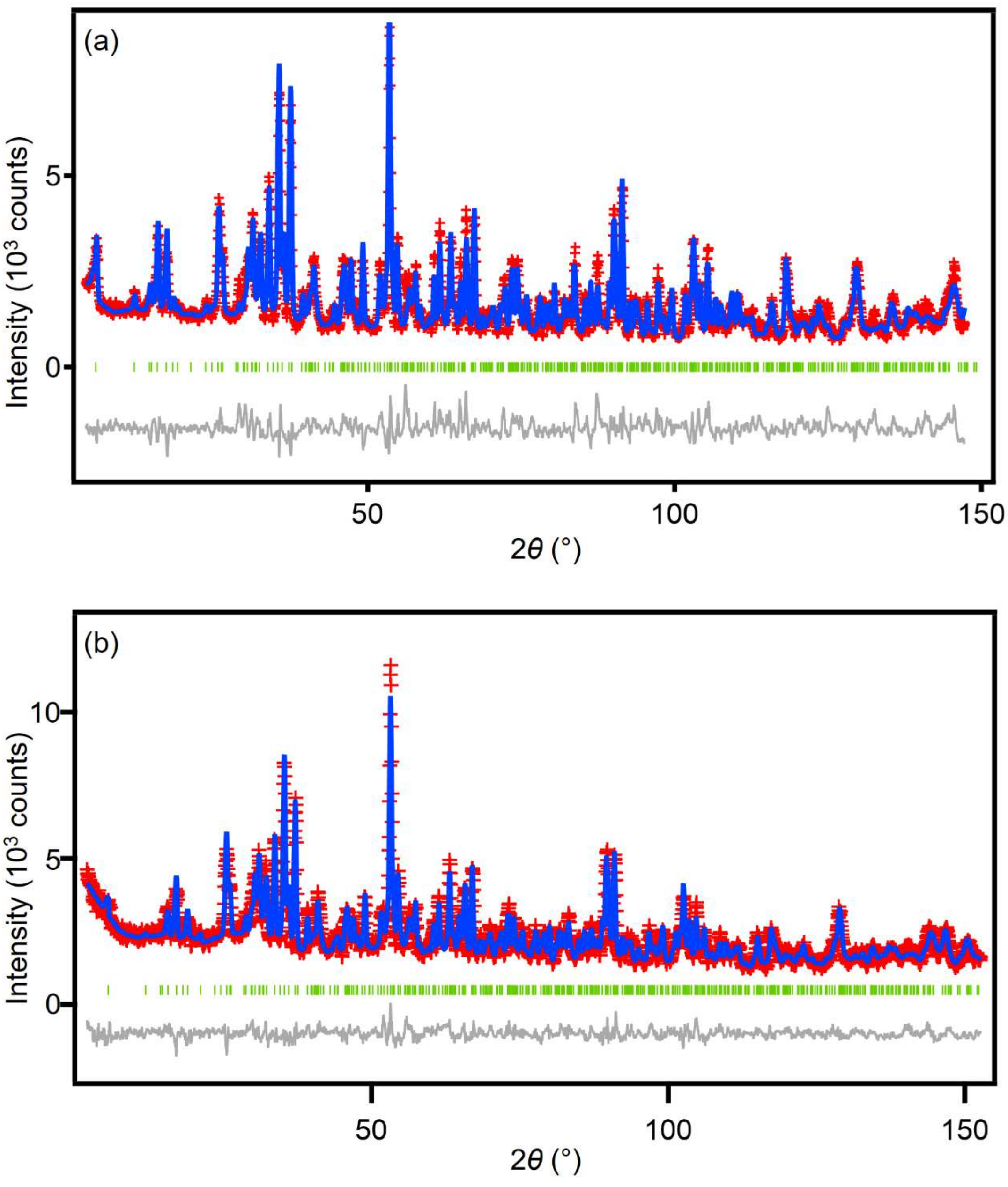

Figure S17. Rietveld patterns of (a) dry $Ba_5Er_2Al_2SnO_{13}$ and (b) hydrated (deuterated) $Ba_5Er_2Al_2SnO_{12}(OD)_2$ at 5 K. Red crosses, blue line, and gray line denote observed, calculated, and difference intensities, respectively. Green vertical bars denote calculated Bragg peak positions of the hexagonal phase.

Table S3. Refined crystal parameters and reliability factors in the Rietveld analysis of the neutron diffraction data of dry $Ba_5Er_2Al_2SnO_{13}$ at 5 K.

Site *X*, Atom label *X*	Atom *Y*	Wyckoff position	$g(Y; X)^a$	*x*	*y*	*z*	$U(Y; X)^b$	BVS^c
Ba1	Ba	4*e*	1	0	0	0.3622(3)	0.0006(5)	1.64
Ba2	Ba	4*f*	1	2/3	1/3	0.5428(3)	= *U*(Ba1)	1.79
Ba3	Ba	6*h*	1/3	0.615(2)	1/2*x*	1/4	= *U*(Ba1)	1.78
Sn	Sn	2*a*	1	0	0	0	0.0003(3)	3.69
Er	Er	4*f*	1	2/3	1/3	0.39593(18)	= *U*(Sn)	3.41
Al	Al	4*f*	1	2/3	1/3	0.8171(4)	= *U*(Sn)	2.90
O1	O	12*k*	1	0.500(3)	2*x*	0.66079(18)	0.0327(12)	1.73
O2	O	12*k*	1	0.3319(10)	1/2*x*	0.45111(14)	0.0078(5)	2.00
O3	O	6*h*	1/3	0.2925(15)	2*x*	1/4	0.016(4)	1.94

Crystal system: hexagonal; Space group: $P6_3/mmc$; Number of formula units per unit cell: Z = 2. Lattice parameters: $a = b$ = 5.9252(2) Å, c = 24.58365(10) Å. Reliability factors: R_{wp} = 11.87%, R_p = 9.16%, R_B = 6.85%, R_F = 3.46%.

^{a} $g(Y; X)$: Occupancy factor of atom *X* at the site *Y*. Occupancies were fixed to 1 at the Ba1, Ba2, Sn, Er, Al, O1, and O2 sites, and fixed to 1/3 at the Ba3 and O3 sites. In a preliminary analysis, the occupancy factors were found to be unity at all the cation sites and at the O1, O2, and O3 sites. The occupational disorder of the Er, Al, and Sn atoms did not improve the refinement results, indicating the occupational order of Er, Al, and Sn atoms.

^{b} $U(Y; X)$: Isotropic atomic displacement parameter of atom *Y* at the site *X*.

^{c} BVS: Bond valence sum. BVSs of Ba, Sn, Er, Al, and O atoms were calculated using the bond-valence parameters reported in the literature.[11]

In a preliminary analysis, the occupancy factor of the interstitial oxygen atom O4 at the 2*b* (0, 0, 1/4) site in the h′ layer was refined to be 0.02(2). Therefore, the occupancy factor of the interstitial oxygen atom was fixed to zero, indicating that the dry sample $Ba_5Er_2Al_2SnO_{13}$ has neither interstitial oxygen atoms nor extra oxygen atoms due to the hydration.

Table S4. Refined crystal parameters and reliability factors in the Rietveld analysis of the neutron diffraction data of hydrated $Ba_5Er_2Al_2SnO_{12}(OD)_2$ (= $Ba_5Er_2Al_2SnO_{13}\cdot D_2O$ = $Ba_5Er_2Al_2SnO_{14}D_2$) at 5 K.

Site *X* Atom label *X*	Atom *Y*	Wyckoff position	$g(Y; X)^a$	*x*	*y*	*z*	$U(Y; X)^b$	BVS^c
Ba1	Ba	4*e*	1	0	0	0.3605(2)	0.0040(6)	1.89
Ba2	Ba	4*f*	1	2/3	1/3	0.5416(2)	= *U*(Ba1)	1.79
Ba3	Ba	6*h*	1/3	0.621(3)	1/2*x*	1/4	= *U*(Ba1)	1.78
Sn	Sn	2*a*	1	0	0	0	0.00035(10)	3.69
Er	Er	4*f*	1	2/3	1/3	0.39948(15)	=*U*(Sn)	3.41
Al	Al	4*f*	1	2/3	1/3	0.8170(3)	=*U*(Sn)	2.90
O1	O	12*k*	1	0.505(2)	2*x*	0.65853(18)	0.0241(12)	1.73
O2	O	12*k*	1	0.3264(8)	1/2*x*	0.45121(11)	0.0060(4)	2.00
O3	O	6*h*	1/3	0.3037(14)	2*x*	1/4	0.021(4)	1.94
O4	O	2*b*	1^d	0	0	1/4	0.168(13)	1.43
D1	D	6*h*	$0.315(10)^e$	0.213(6)	1/2*x*	1/4	0.094(9)	0.91
D2	D	12*k*	$0.175(5)^e$	0.210(6)	1/2*x*	0.4138(8)	= *U*(D1)	0.81

Crystal system: hexagonal; Space group: $P6_3/mmc$; Number of formula units per unit cell: Z = 2. Lattice parameters: $a = b$ = 5.94505(18) Å, c = 24.6890(9) Å. Reliability factors: R_{wp} = 6.44%, R_p = 5.13%, R_B = 3.53%, R_F = 1.72%.

^{a} $g(Y; X)$: Occupancy factor of *Y* atom at the *X* site. The occupancy factors were fixed to 1 at the Ba1, Ba2, Sn, Er, Al, O1, and O2 sites, and fixed to 1/3 at the Ba3 and O3 sites.

^{b} $U(Y; X)$: Isotropic atomic displacement parameter of atom *Y* at the site *X*.

^{c} BVS: Bond valence sum. The BVSs of Ba, Sn, Er, Al, O, and D atoms were calculated using the bond-valence parameters reported in the literature.[11,12]

^{d} In preliminary analyses, the reliability factor for the structural model with the O4 atom was R_{wp} = 8.96%, which was much lower than that without the O4 atom, R_{wp} = 11.03%. The occupancy factor of the oxygen atoms at the O4 site was refined to g(O; O4) = 1.08(3) in another preliminary analysis. Therefore, in the final refinement, the g(O; O4) was fixed to unity (g(O; O4) = 1.000), which was also validated by the variation of the reliability factor R_{wp} with the occupancy factor g(O; O4) (**Figure S18**). The value g(O; O4) = 1.000 was in agreement with the TG data.

^{e} In a preliminary analysis, the sum of the number of deuteron atoms in a unit cell for $Ba_5Er_2Al_2SnO_{13-5y/2}(OD)_{5y}$ was refined to be $6g(D; D1) + 12g(D; D2)$ = 4.20(3), which was higher than that (4.00) for $Ba_5Er_2Al_2SnO_{12}(OD)_2$. Here $g(X; Xn)$ is the occupancy factor of the *X* atom at the *Xn* site. Therefore, the sum of deuteron atoms in a unit cell was fixed to 4: $6g(D; D1) + 12g(D; D2) = 4$.

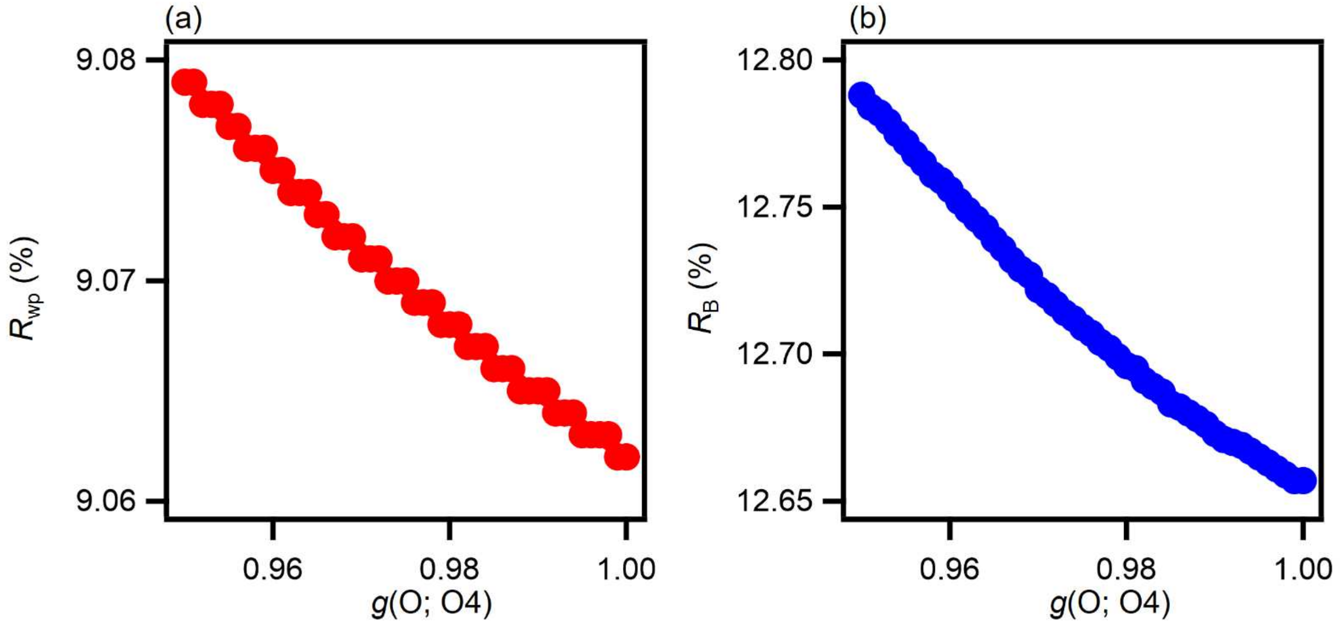

Figure S18. Variation of the reliability factors (a) R_{wp} (red circles) and (b) R_B (blue circles) in the Rietveld analyses for the fixed occupancy factor *g*(O; O4) step-by-step (0.001 step interval) using the neutron diffraction data of hydrated (deuterated) $Ba_5Er_2Al_2SnO_{13}\cdot D_2O$ at 5 K, validating the *g*(O; O4) value of 1.000.

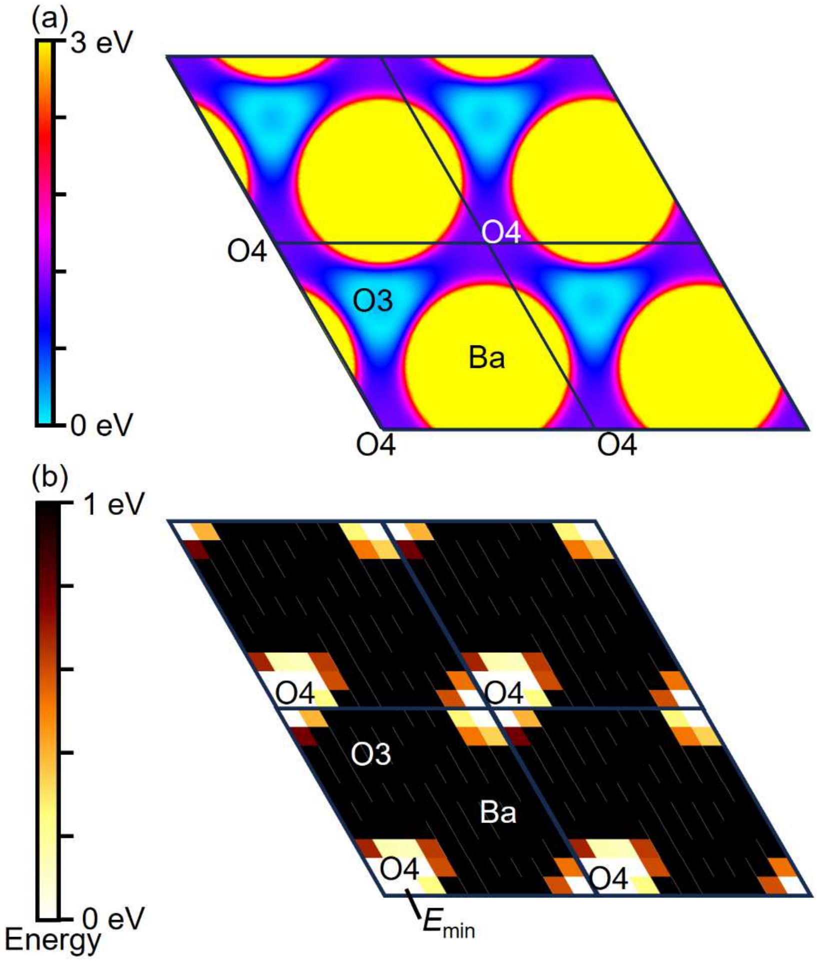

Figure S19. (a) Bond-valence-based energy landscape of $Ba_5Er_2Al_2SnO_{13}$ for a test oxide ion on the *ab* plane at $z = 1/4$ obtained using the crystal parameters in **Table S3**. (b) Total energy landscape of $Ba_5Er_2Al_2SnO_{14}$ including an additional oxygen atom placed on the *ab* plane at $z = 1/4$, which was obtained by structural optimization through the static DFT calculations. E_{min} denotes the minimum energy position, which was set to 0 eV. These figures show that the only possible oxygen sites on the *ab* plane at $z = 1/4$ are the O3 and O4 sites.

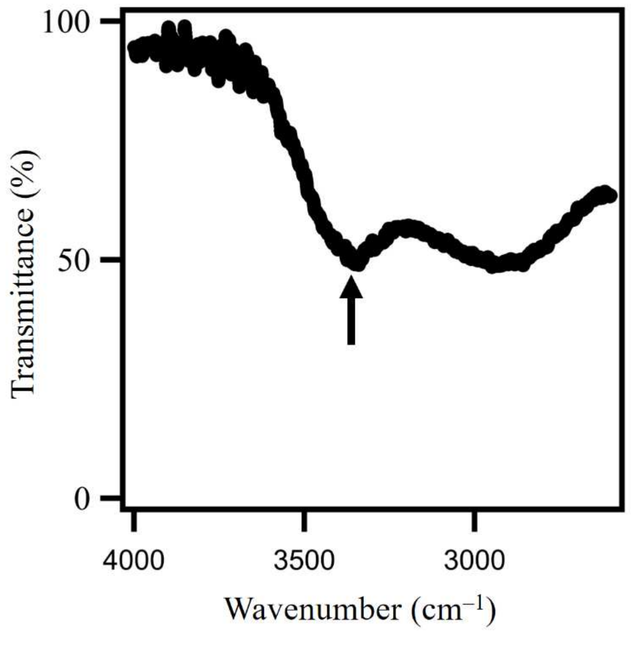

Figure S20. IR spectrum of BEAS. Using an empirical equation between OH bond length and IR frequency, the intramolecular OH bond length in BEAS was calculated to be 0.99(4) Å.[13]

Supplementary Note 5. Crystal structures of $Ba_5Er_2Al_2SnO_{13}$ and $Ba_5Er_2Al_2SnO_{12}(OD)_2$.

Here, we describe the refined crystal structures of dry BEAS ($Ba_5Er_2Al_2SnO_{13}$) and hydrated (deuterated) BEAS [$Ba_5Er_2Al_2SnO_{12}(OD)_2$] obtained by the Rietveld analyses of neutron diffraction data at 5 K (**Figure 3**). The crystal structure of $Ba_5Er_2Al_2SnO_{13}$ and $Ba_5Er_2Al_2SnO_{12}(OD)_2$ shows stacking of eight cubic close-packed BaO_3 (c) layers [four (Ba1)$(O1)_3$ layers and four (Ba2)$(O2)_3$ layers] and two intrinsically oxygen-deficient hexagonal close-packed $BaO_{1+\varepsilon}$ (h′) layers in the order of $(cch'cc)_2$. Here the ε is the occupancy factor of oxygen atoms at the interstitial O4 oxygen site of *g*(O; O4) where ε = 0 in $Ba_5Er_2Al_2SnO_{13}$ and ε = 1.00 in $Ba_5Er_2Al_2SnO_{13}\cdot D_2O$. The crystal structure of $Ba_5Er_2Al_2SnO_{13}$ consists of eight c BaO_3 and two h′ BaO layers, Er, Al, and Sn atoms (**Figure 3a**), while the structure of $Ba_5Er_2Al_2SnO_{12}(OD)_2$ consists of eight c BaO_3 and two h′ BaO_2 layers, Er, Al, Sn, and D atoms (**Figure 3b**).

In both $Ba_5Er_2Al_2SnO_{13}$ and $Ba_5Er_2Al_2SnO_{12}(OD)_2$, two ErO_6 octahedra and one SnO_6 octahedron are connected by corner-sharing O2 atoms, to form [ErO_6–SnO_6–ErO_6] triple octahedral layers [octahedral trimer $(Er_2Sn)O_{16}$], and two AlO_4 tetrahedra are connected by one corner-sharing O3 atom to form an Al_2O_7 dimer. The crystal structure of $Ba_5Er_2Al_2SnO_{13}$ and $Ba_5Er_2Al_2SnO_{12}(OD)_2$ has two [ErO_6–SnO_6–ErO_6] triple octahedral layers and two Al_2O_7 double tetrahedral layers. An Al_2O_7 double tetrahedral layer has no interstitial O4 atoms in $Ba_5Er_2Al_2SnO_{13}$, while an Al_2O_7 double tetrahedral layer contains one interstitial O4 atom in $Ba_5Er_2Al_2SnO_{12}(OD)_2$. The crystal structure of $Ba_5Er_2Al_2SnO_{13}$ consists of ErO_6 and SnO_6 octahedra, Al_2O_7 dimers, and Ba atoms (**Figure 3a**), while the structure of $Ba_5Er_2Al_2SnO_{12}(OD)_2$ consists of ErO_6 and SnO_6 octahedra, Al_2O_7 dimers, Ba, O4, and D atoms (**Figure 3b**).

The structure of $Ba_5Er_2Al_2SnO_{13}$ is also considered to consist of two [ErO_6–SnO_6–ErO_6] triple octahedral layers and two h′ BaO layers, and Al atoms (**Figure 3a**), while the structure of $Ba_5Er_2Al_2SnO_{12}(OD)_2$ is considered to consist of two [ErO_6–SnO_6–ErO_6] triple octahedral layers and two h′ BaO_2 layers, Al and D atoms (**Figure 3b**).

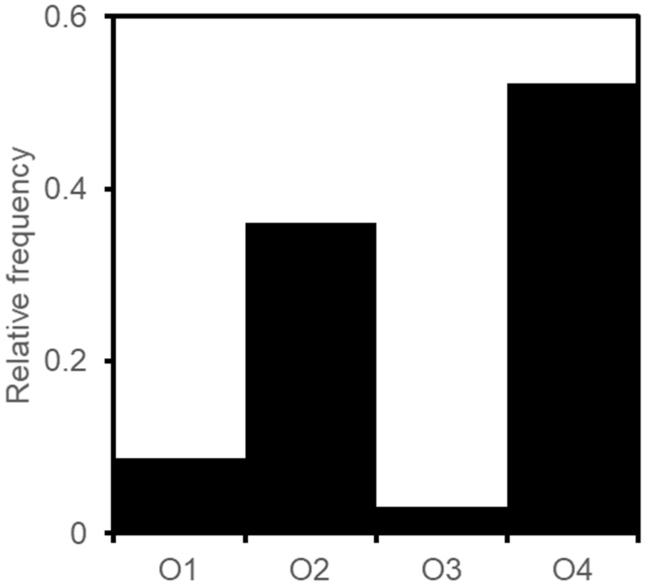

Figure S21. Relative frequencies of oxygen atoms nearest to each proton in $Ba_{40}Er_{16}Al_{16}Sn_8O_{112}H_{16}$ for the AIMD simulations at 1200 °C. Protons were mainly located near the O4 and O2 atoms.

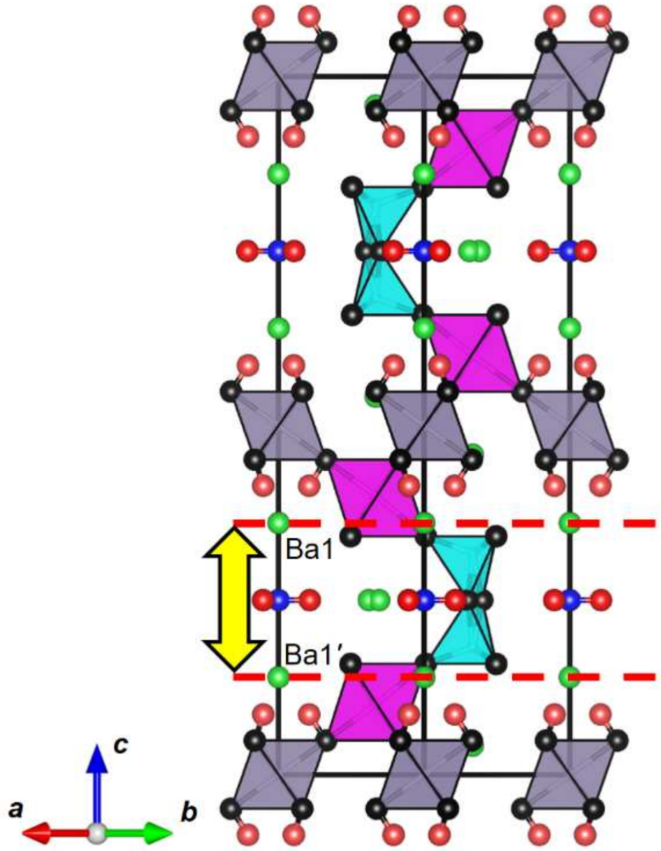

Figure S22. The thickness of the h′ layer in BEAS (yellow arrow in **Figure S22**). The thickness of the h′ layer was defined by the interatomic distance between the Ba1 and Ba1′ atoms:

$c(z(\mathrm{Ba1}) - z(\mathrm{Ba1}')) = c(2z(\mathrm{Ba1}) - 1/2)$,

where $z(X)$ is the z atomic coordinates of X atom. Here, the Ba1 is the Ba1 atom at the atomic coordinates of $1 + x, y, z$, and Ba1′ is the Ba1 atom at $1 + x, y, -z+1/2$, where the values of x, y, and z for dry and hydrated BEAS are listed in **Tables S3** and **S4**, respectively.

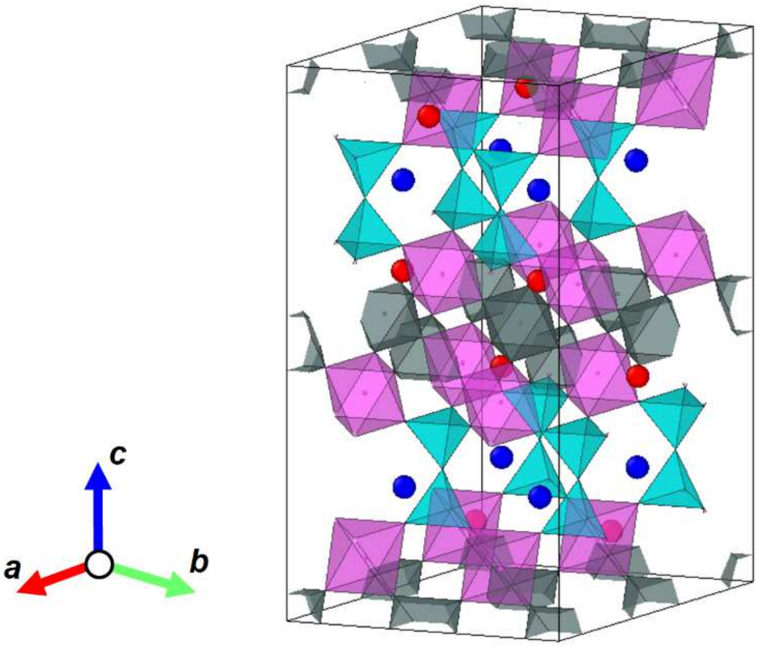

Figure S23. Optimized structure of 2×2×1 supercell ($Ba_{40}Er_{16}Al_{16}Sn_8O_{112}H_{16}$) used as the initial structure in AIMD simulations. Pink, gray, and light blue polyhedra represent ErO_6 and SnO_6 octahedra, and AlO_4 tetrahedra, respectively. Red and blue spheres represent H atoms located near lattice oxygen O2 atoms in octahedral layers and interstitial oxygen O4 atoms in the h′ layers, respectively.

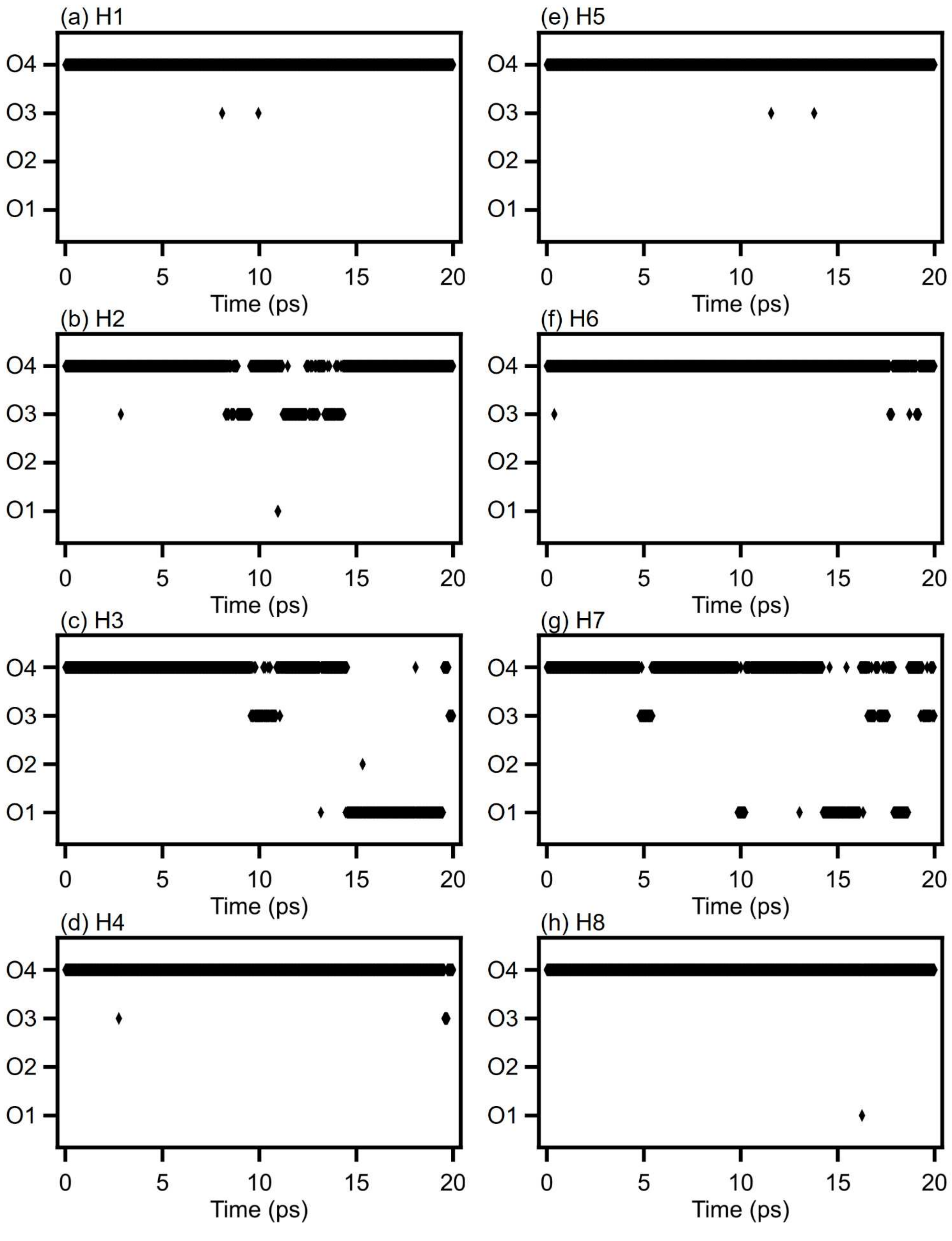

Figure S24. Time evolution of the O1–O4 oxygen atoms closest to (a) H1, (b) H2, (c) H3, (d) H4, (e) H5, (f) H6, (g) H7, and (h) H8 obtained from the AIMD simulations at 1200 °C.

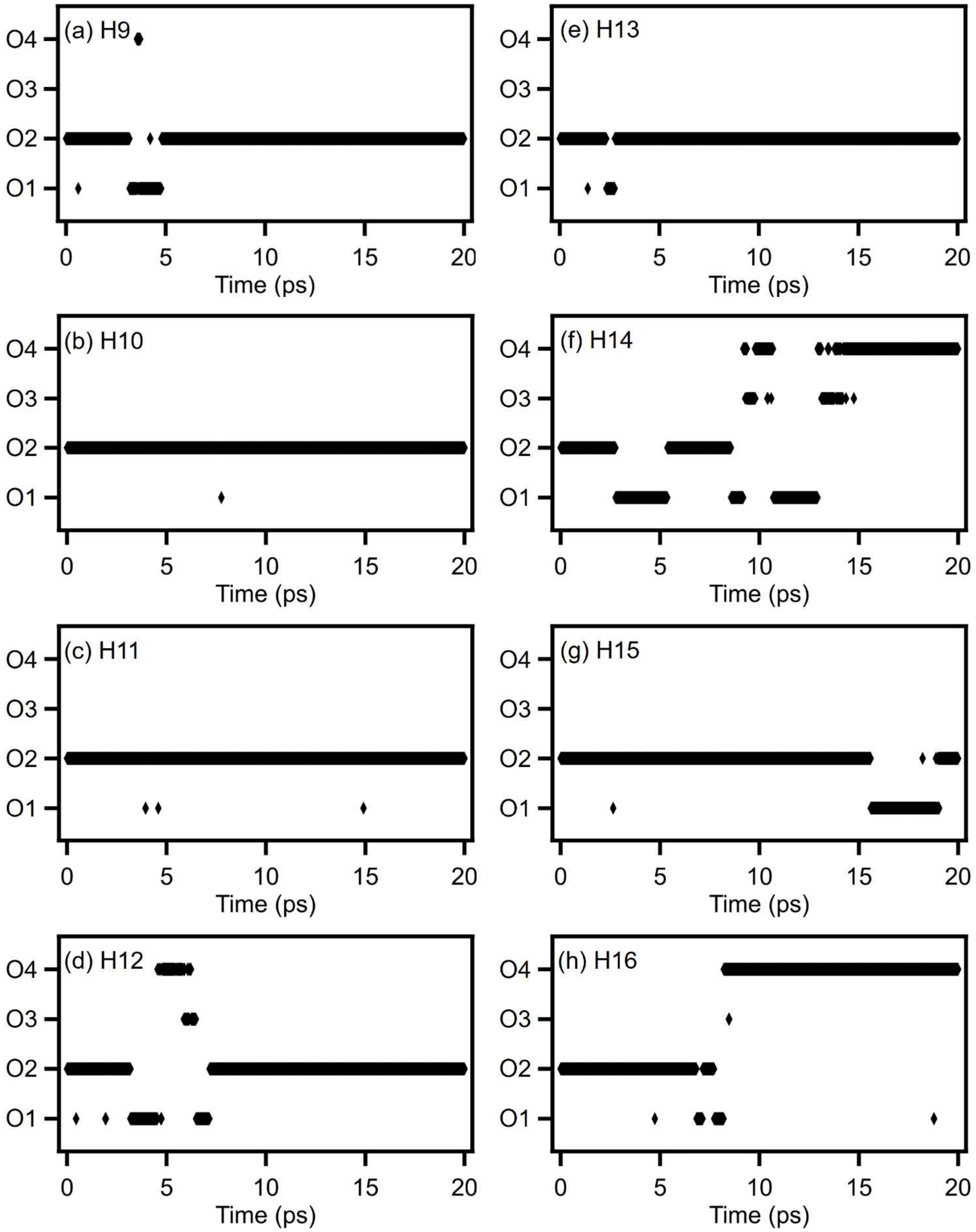

Figure S25. Time evolution of the O1–O4 oxygen atoms closest to (a) H9, (b) H10, (c) H11, (d) H12, (e) H13, (f) H14, (g) H15, and (h) H16 obtained from the AIMD simulations at 1200 °C.

Supplementary Note 6. Migration of the protons H3, H7, H14, and H16 as studied by the time evolution of the oxygen atoms closest to each proton in the AIMD simulations of $Ba_{40}Er_{16}Al_{16}Sn_8O_{112}H_{16}$ at 1200 °C.

In this Note 6, we describe the details of the complicated behavior of proton migration in the AIMD simulations of $Ba_{40}Er_{16}Al_{16}Sn_8O_{112}H_{16}$ at 1200 °C. In the AIMD simulations, the H1–H8 atoms were placed in the h′ layers in the initial structure. Each proton of H1, H2, H4–H6, and H8 was mainly located closest to an O4 atom (**Figures S24a**, **b**, **d–f**, and **h; Figure 5a, b,** and **d**), indicating the trapping of these H atoms by O4 atoms. On the contrary, the H3 atom moved from the sites closest to the O4 atom to the sites near the O1 atom as follows (**Figure S24c**), which will be described simply as "the H3 atom moved from the O4 atom to the O1 atom". The H3 atom was closest to the O4 atom at 0–9.5 ps, near the O3 atom at 9.5–10.9 ps, and closest to the O4 atom at 10.9–14.5 ps. At 14.5 ps, the H3 atom migrated from the O4 atom to the O1 atom, and remained near the O1 atom until 19.5 ps, indicating that the H3 atom migrated from the O4 in the h′ layer to the O1 near the interface between the h′ and octahedral layers. The H7 atom moved back and forth between the O4 in the h′ layer and the O1 atom near the interface between the h′ and octahedral layers as follows (**Figure S24g**). The H7 atom was closest to the O4 atom during 0–4.8 ps, near the O3 atom at 4.8–5.5 ps, closest to the O4 atom again at 5.5–9.8 ps, near the O1 atom at 9.8–10.2 ps, and closest to the O4 atom again from 10.2 ps to 14.5 ps. The H7 atom migrated from the O4 atom to the O1 atom at 14.5 ps and was near the O1 atom between 14.5 ps to 16 ps. The H7 moved back and forth between the O3 and O4 atoms from 16 ps to 17.8 ps. At 17.8 ps, the H7 atom migrated from the O4 atom to O1 atom and was near the O1 atom between 17.8 ps to 18.6 ps. Then, the H7 moved back and forth between the O3 and O4 atoms at 18.6–20 ps. Thus, each proton of the H3 and H7 moved back and forth among the O4, O1 and O3 atoms in the h′ layer, although these H3 and H7 were mainly closest to the O4 (**Figure S24c**, **g**). On the one hand, other protons of H1, H2, H4–H6, and H8 were mainly located closest to an O4 atom (**Figures S24a**, **b**, **d–f**, and **h; Figure 5a, b,** and **d**), indicating the trapping of these H atoms by O4 atoms.

In the AIMD simulations, the H9–H16 atoms were placed at the sites near the O2 atoms in the octahedral layers in the initial structure. Each proton of H9–H13 and H15 exhibited long-range migrations in the octahedral layers by rotating around the O2 atoms and hopping from one O2 atom to the nearest-neighbor O2 atom (**Figures S25a–e,** and **g; Figure S26; Figure 5a, c, e,** and **f).** In contrast, the H14 atom migrated from the O2 atom in the octahedral layer to the O4 atom in the h′ layer through the O1 atom at the interface between the h′ and octahedral layers (**Figure S25f**). The H14 atom migrated from one O2 atom to a nearest-neighbor O2 atom in the octahedral layer between 0 ps and 2.7 ps and migrated from the O2 to the O1 at 2.7 ps. Then, the H14 atom migrated from the O1 to O2 at 5.5 ps. In the octahedral layer, the H14 atom migrated through four O2 atoms between 5.5 ps and 8.5 ps, and migrated from the O2 to O1 at 8.5 ps. The H14 migrated from the O2 atom to O4 atom via the O1 atom between 8.5 and 9.2 ps. The H14 atom moved back and forth between the O4 and O3 atoms at 9.2–10.7 ps. Then, the H14 atom moved from the O4 to O1 at 10.7 ps, and was near the O1 atom at 10.7–12.9 ps. The H14 atom moved from the O1 to the O4 atom, and then moved back and forth between the O4 and O3 atoms at 12.9–14.3 ps and was localized closest to the O4 atom between 14.3 and 20 ps. In many cases, the H16 atom moved from an O2 atom to a nearest-neighbor O2 atom in the octahedral layer between 0 ps and 7.7 ps (**Figure S25h**). The H16 atom moved through four O2 atoms in the octahedral layer between 7.7 and 8.2 ps. The H16 atom moved from one O2 atom to one O4 atom, and remained closest to the O4 atom at 8.2–20 ps. Thus, the H14 and H16 moved from the O2 atom to the O4 atom (**Figure S25f, h**), while other protons H9–H13 and H15 showed long-range migration through the O2 atoms in the octahedral layers (**Figures S25a–e,** and **g; Figure 5a, c, e,** and **f**).

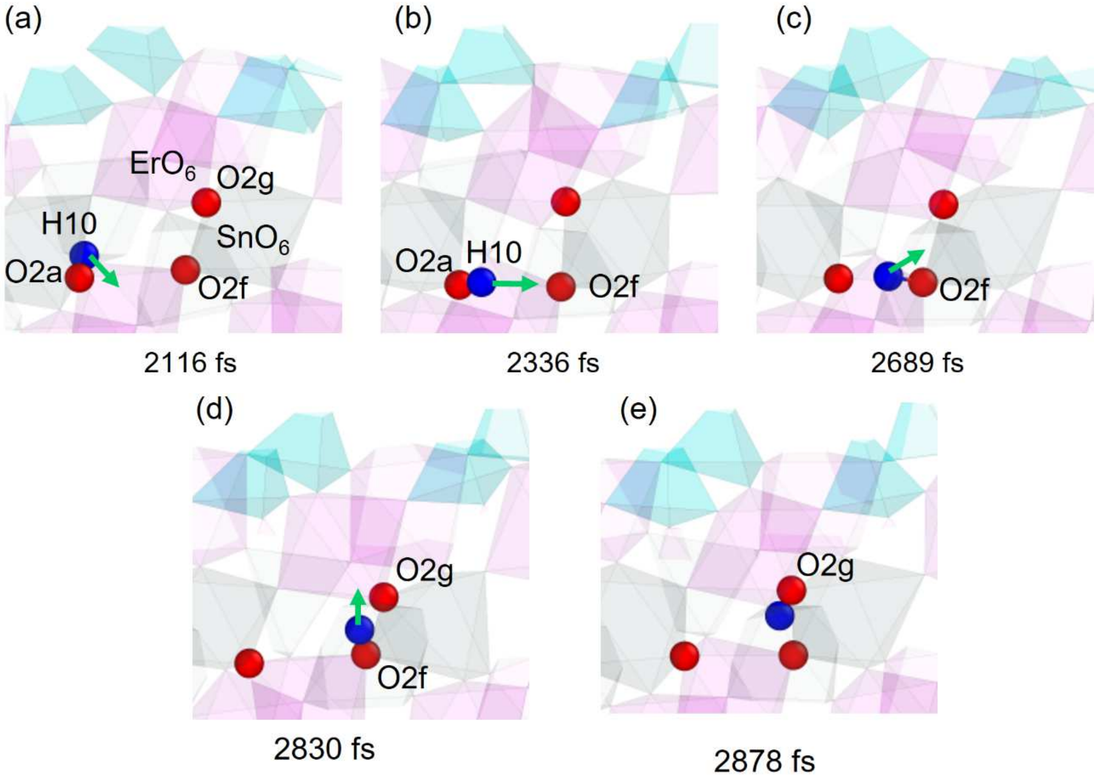

Figure S26. Snapshots of the proton diffusion process of $Ba_{40}Er_{16}Al_{16}Sn_8O_{112}H_{16}$ at 1200 °C. Elapsed times: (a) 2116, (b) 2336, (c) 2689, (d) 2830, and (e) 2878 fs. The red and blue spheres represent the oxygen and hydrogen atoms, respectively. The pink, gray, and light blue polyhedra represent ErO_6 octahedra, SnO_6 octahedra, and AlO_4 tetrahedra, respectively. The green arrows denote the directions to which the proton H10 will move in the next step. At 2116 fs (panel a), the H10 was coordinated to O2a. The O2a migrated through the following steps: reorientation around O2a (from panel a to b), hopping from O2a to O2f (from panel b to c), and reorientation around O2f (from panel c to d) followed by hopping from O2f to O2g (from panel d to e). See also the proton diffusion process in **Video S1**.

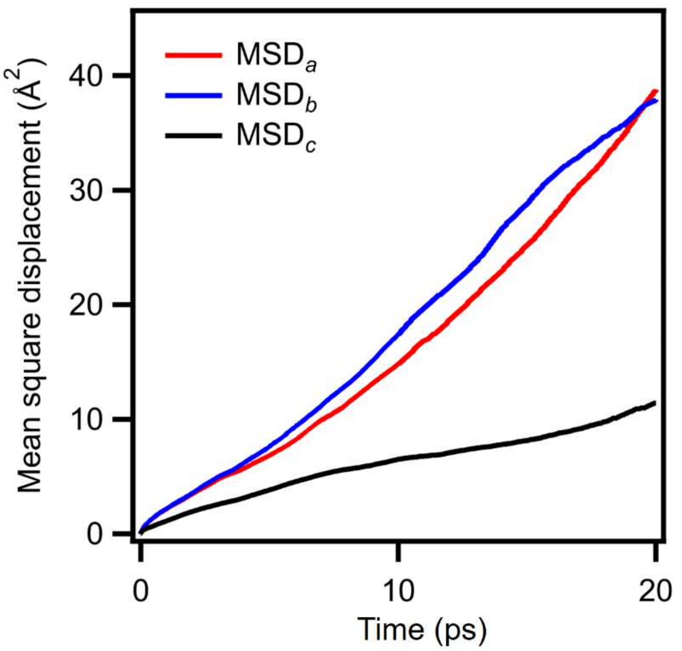

Figure S27. Time dependence of the MSDs of protons in $Ba_{40}Er_{16}Al_{16}Sn_8O_{112}H_{16}$ (= 8 $Ba_5Er_2Al_2SnO_{13}\cdot H_2O$) at 1200 °C. The red, blue, and black curves represent the protons along the *a* (MSD_a), *b* (MSD_b), and *c* (MSD_c) axes, respectively. The MSD_a and MSD_b were higher than the MSD_c (e.g., 3.3 times higher at 20 ps), indicating two-dimensional (2D) proton migration. Similar 2D proton diffusion in the octahedral layers was also reported in other hexagonal perovskite-related oxides.[5, 9, 14–16]

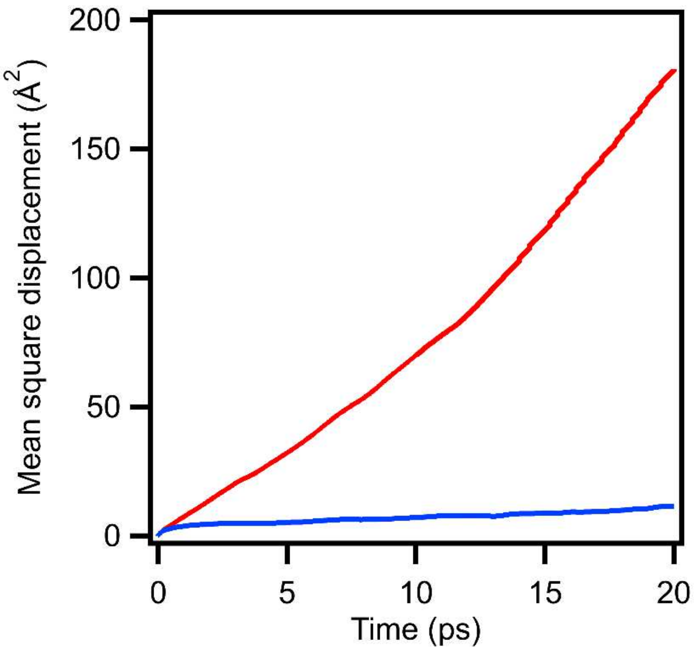

Figure S28. Time dependence of the MSDs of protons in $Ba_{40}Er_{16}Al_{16}Sn_8O_{112}H_{16}$ (= 8 $Ba_5Er_2Al_2SnO_{13}\cdot H_2O$) at 1200 °C. The blue and red curves represent the protons H1–H8 and H9–H16, respectively. In the initial structure, the eight protons H1–H8 were located in the h′ layers, and the other eight protons H9–H16 were located in the triple-octahedral layers (**Figure S23**).

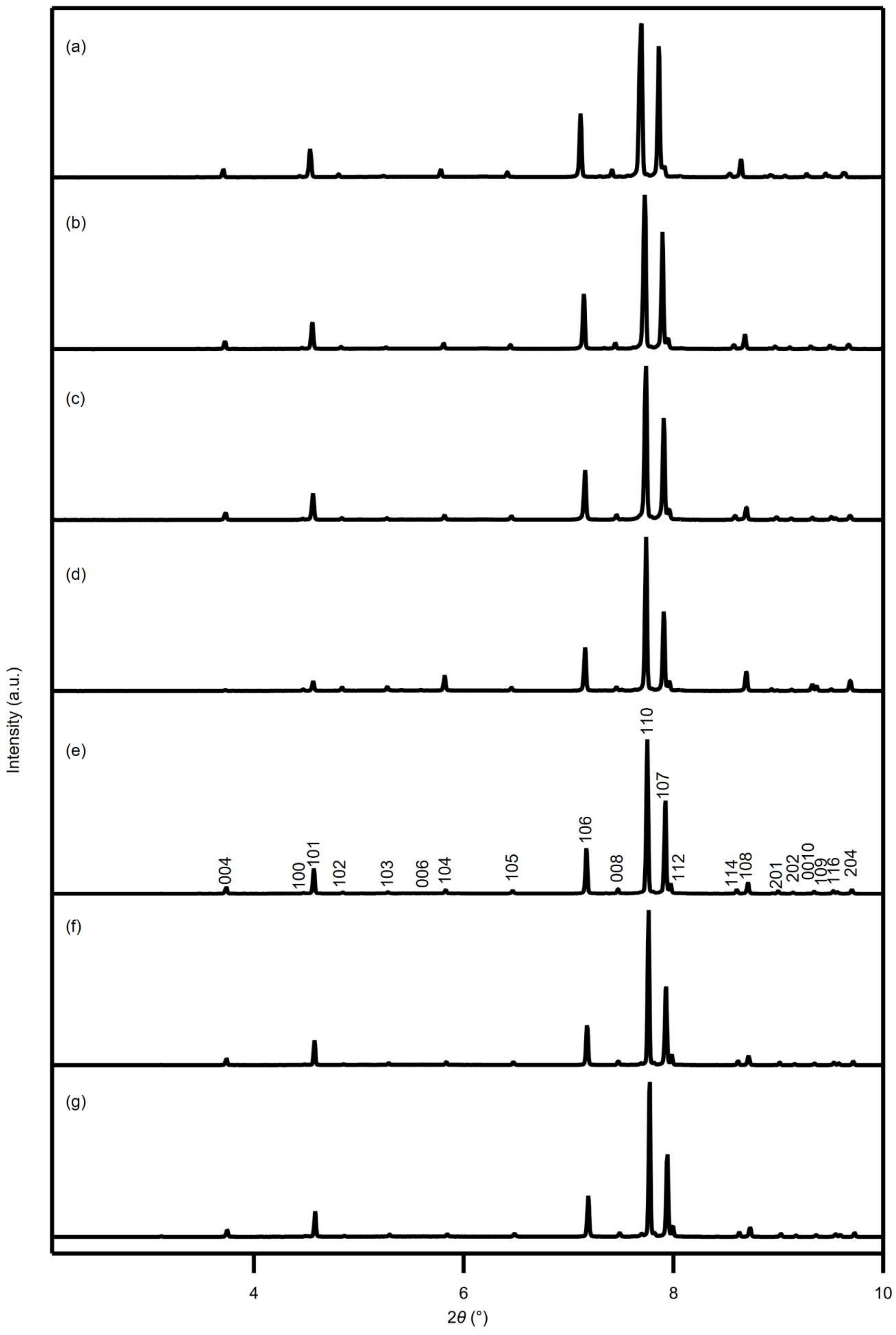

Figure S29. Synchrotron XRD patterns of as-prepared $Ba_5R_2Al_2SnO_{13-5y/2}\cdot(OH)_{5y}$ (R = (a) Gd^{3+}, (b) Dy^{3+}, (c) Ho^{3+}, (d) Y^{3+}, (e) Er^{3+}, (f) Tm^{3+}, (g) Yb^{3+}) in static air. The X-ray wavelength was 0.4006422(7) Å. *hkl* denotes the reflection index of the hexagonal phase.

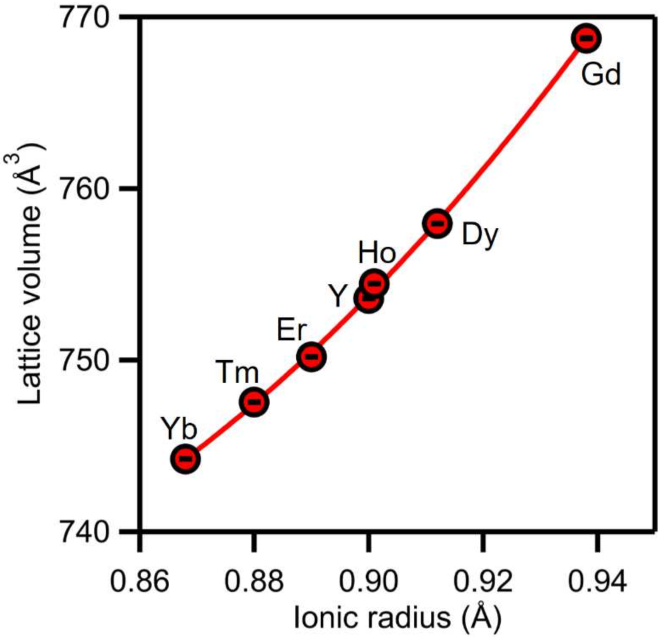

Figure S30. Lattice volume of as-prepared $Ba_5R_2Al_2SnO_{13-5y/2}\cdot(OH)_{5y}$ versus ionic radius of *R* cation for coordination number of 6 (Ref. [16]) at 27 °C (*R* = Yb^{3+}, Tm^{3+}, Er^{3+}, Y^{3+}, Ho^{3+}, Dy^{3+}, and Gd^{3+}). The lattice volume of as-prepared $Ba_5R_2Al_2SnO_{13-5y/2}\cdot(OH)_{5y}$ increases with increasing of the ionic radius of the *R* cation.

Table S5. Lattice parameters of as-prepared $Ba_5R_2Al_2SnO_{13-5y/2}\cdot(OH)_{5y}$ (*R* = Yb^{3+}, Tm^{3+}, Er^{3+}, Y^{3+}, Ho^{3+}, Dy^{3+}, and Gd^{3+}), which were refined using synchrotron XRD data at 27 °C.

R in $Ba_5R_2Al_2SnO_{13-5y/2}\cdot(OH)_{5y}$	Ionic radius for coordination number of 6 (Å) (Ref.[17])	*a*, *b* (Å)	*c* (Å)	Lattice volume (Å^3)
Gd^{3+}	0.938	5.98204(17)	24.8058(4)	768.74(2)
Dy^{3+}	0.912	5.95275(11)	24.6989(3)	757.957(14)
Ho^{3+}	0.901	5.94389(9)	24.6581(2)	754.452(12)
Y^{3+}	0.9	5.94066(7)	24.6567(2)	753.589(9)
Er^{3+}	0.89	5.93266(6)	24.61157(18)	750.187(9)
Tm^{3+}	0.88	5.92336(6)	24.60224(18)	747.552(8)
Yb^{3+}	0.868	5.91531(7)	24.55976(19)	744.236(9)

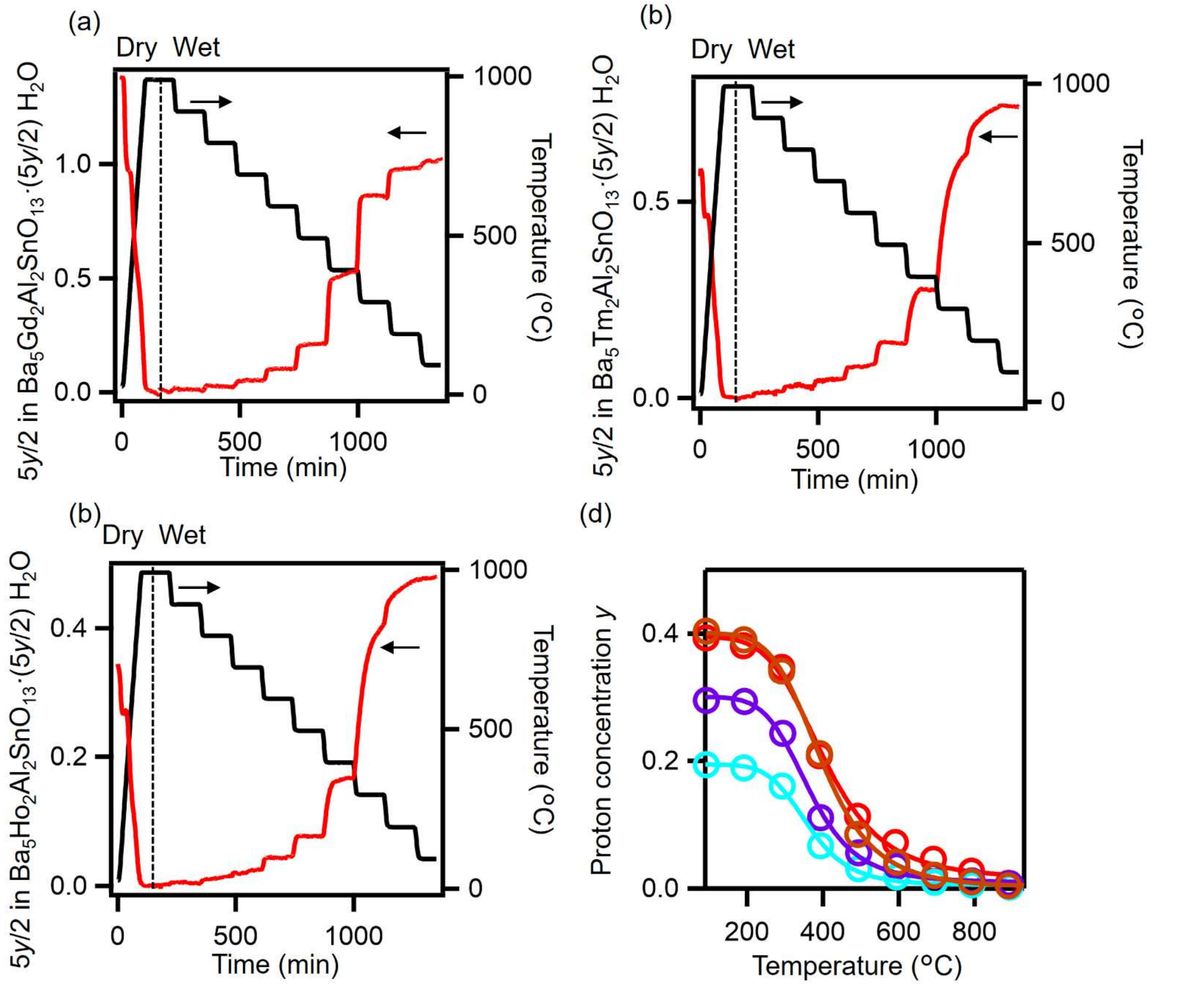

Figure S31. Water uptake of $Ba_5R_2Al_2SnO_{13}\cdot(5y/2)$ H_2O (R = Tm, Gd, and Ho). (a)–(c) TG results of (a) $Ba_5Gd_2Al_2SnO_{13}\cdot(5y/2)$ H_2O, (b) $Ba_5Tm_2Al_2SnO_{13}\cdot(5y/2)$ H_2O, and (c) $Ba_5Ho_2Al_2SnO_{13}\cdot(5y/2)$ H_2O. Time dependencies of temperature (black line) and the amount of water $5y/2$ in $Ba_5R_2Al_2SnO_{13}\cdot(5y/2)$ H_2O (red line) in dry N_2 (time < 160 min) and wet N_2 (time > 160 min; water vapor partial pressure of 0.021 atm). The amount of water $5y/2$ in $Ba_5R_2Al_2SnO_{13}\cdot(5y/2)$ H_2O was estimated by assuming that the $5y/2$ is zero at 1000 °C in dry N_2. (d) Temperature dependence of the proton concentration y in $Ba_5R_2Al_2SnO_{13}\cdot(5y/2)$ H_2O (R = Tm, purple open circles and line, y = 0.29, F_w = 0.73 at 100 °C; R = Er, red open circles and line, y = 0.4, F_w = 1.0 at 100 °C; R = Gd, brown open circles and line, y = 0.4, F_w = 1.0 at 100 °C; R = Ho, light blue open circles and line, y = 0.19, F_w = 0.48 at 100 °C). The proton concentration y and the fractional water uptake F_w of $Ba_5R_2Al_2SnO_{13}\cdot(5y/2)$ H_2O depend on the R species.

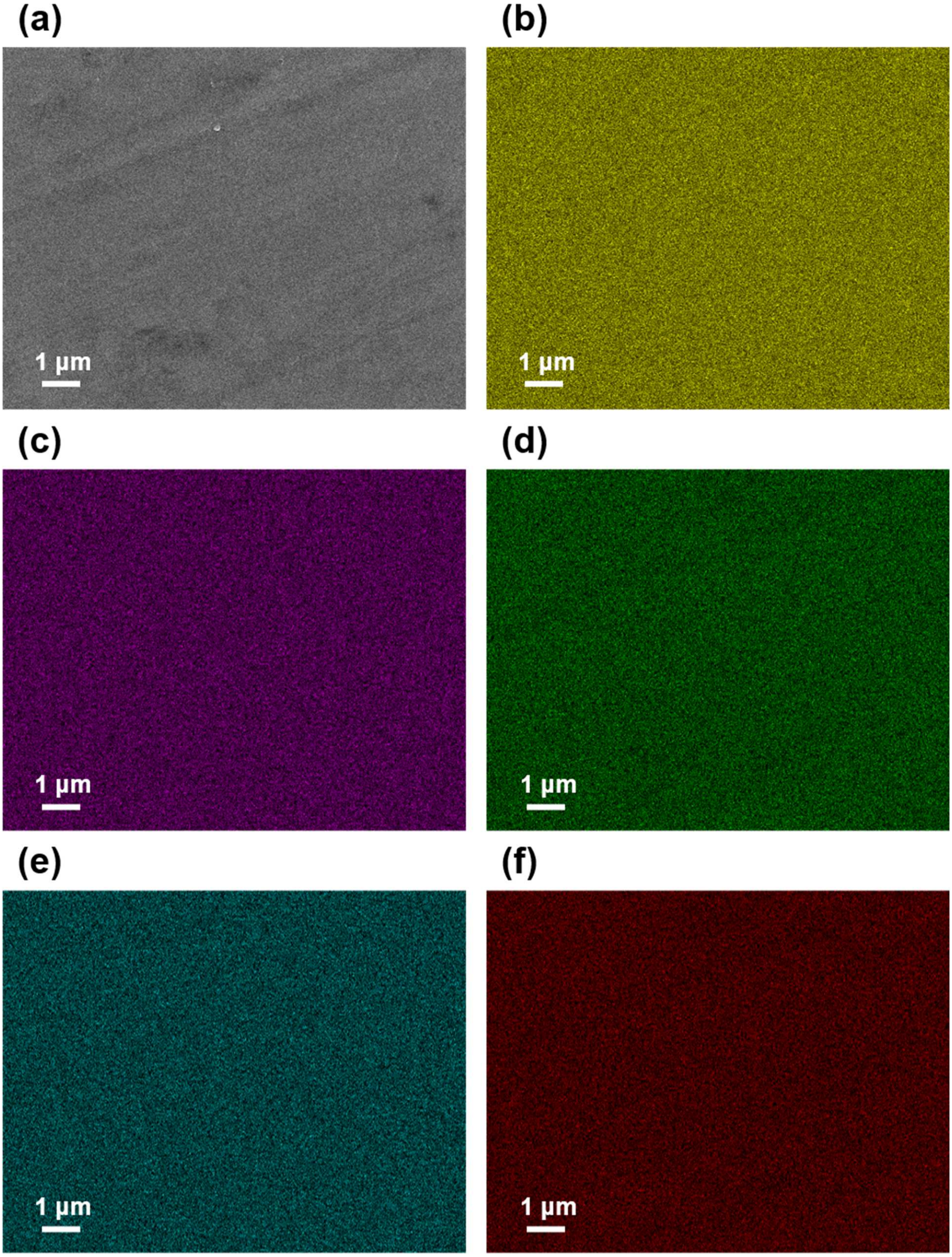

Figure S32. (a) SEM micrograph and EDS elemental mapping of (b) Ba, (c) Er, (d) Al, (e) Sn, and (f) O elements of BEAS.

Reference

(1) Paparazzo, E. XPS Analysis of Iron Aluminum Oxide Systems. *Appl. Surf. Sci.* **1986**, *25*, 1–12.

(2) Kwoka, M.; Ottaviano, L.; Passacantando, M.; Santucci, S.; Czempik, G.; Szuber, J. XPS Study of the Surface Chemistry of L-CVD SnO_2 Thin Films after Oxidation. *Thin Solid Films* **2005**, *490* (1), 36–42.

(3) Schmitz, P. J. Characterization of the Surface of $BaCO_3$ Powder by XPS. *Surf. Sci. Spectra* **2001**, *8* (3), 190–194.

(4) Yoon, H. J.; Lee, J.; Kim, Y.-I.; Cho, D. W.; Sohn, Y. Synthesis and Characterization of Er_2O_3 Nanorods and Nanosheets. *Ceram. Int.* **2017**, *43* (2), 2069–2075.

(5) Murakami, T.; Hester, J. R.; Yashima, M. High Proton Conductivity in $Ba_5Er_2Al_2ZrO_{13}$, a Hexagonal Perovskite-Related Oxide with Intrinsically Oxygen-Deficient Layers. *J. Am. Chem. Soc.* **2020**, *142* (27), 11653–11657.

(6) Kreuer, K. D. Proton-Conducting Oxides. *Annu. Rev. Mater. Res.* **2003**, *33* (1), 333–359.

(7) Fop, S.; McCombie, K. S.; Wildman, E. J.; Skakle, J. M. S.; Irvine, J. T. S.; Connor, P. A.; Savaniu, C.; Ritter, C.; Mclaughlin, A. C. High Oxide Ion and Proton Conductivity in a Disordered Hexagonal Perovskite. *Nat. Mater.* **2020**, *19* (7), 752–757.

(8) Saito, K.; Yashima, M. High Proton Conductivity within the 'Norby gap' by Stabilizing a Perovskite with Disordered Intrinsic Oxygen Vacancies. *Nat. Commun.* **2023**, *14*, 7466.

(9) Morikawa, R.; Murakami, T.; Fujii, K.; Nambu, Y.; Yashima, M. High Proton Conduction in Ba_2LuAlO_5 with Highly Oxygen-Deficient Layers. *Commun. Mater.* **2023**, *4*, 42.

(10) Shin, J. F.; Orera, A.; Apperley, D. C.; Slater, P. R. Oxyanion Doping Strategies to Enhance the Ionic Conductivity in $Ba_2In_2O_5$. *J. Mater. Chem.* **2011**, *21* (3), 874–879

(11) Brown, I. D.; Aitermatt, D. Bond-valence parameters obtained from a systematic analysis of the inorganic crystal structure database. *Acta Crystallogr., Sect. B: Struct. Sci.* **1985**, *41* (4), 244–247.

(12) Brown, I. D. *The Chemical Bond in Inorganic Chemistry*, Oxford University Press, Oxford, **2002**.

(13) Novak, A. Hydrogen Bonding in Solids. Correlation of Spectroscopic and Crystallographic Data. *Large Molecules. Berlin, Heidelberg: Springer Berlin Heidelberg,* **1974**, *18*, 177– 216

(14) Murakami, T.; Avdeev, M.; Morikawa, R.; Hester, J. R.; Yashima, M. High Proton Conductivity in β-Ba_2ScAlO_5 Enabled by Octahedral and Intrinsically Oxygen-Deficient Layers. *Adv. Funct. Mater.* **2023**, *33* (7), 2206777.

(15) Sakuda, Y.; Murakami, T.; Avdeev, M.; Fujii, K.; Yasui, Y.; Hester, J. R.; Hagihala, M.; Ikeda, Y.; Nambu, Y.; Yashima, M. Dimer-Mediated Cooperative Mechanism of Ultrafast-Ion of Ultrafast-Ion Conduction in Hexagonal Perovskite-Related Oxides. *Chem. Mater.* **2023**, *35* (22), 9774–9788.

(16) Youn, Y.; Hussain, B.; Ullah, A.; Hwang, I. J.; Shin, J.; Hong, J.; Joh, D. W.; Lee, S.; Song, R.; Park, S.; Kim, T. W.; Choi, Y.; Lim, T.; Kim, H. Anisotropic Proton Migration in Hexagonal Perovskite-Related $Ba_5Er_2Al_2ZrO_{13}$ Oxide. *Chem. Mater.* **2023**, *35* (22), 9493–9504.

(17) Shannon, R. D. Revised Effective Ionic Radii and Systematic Studies of Interatomic Distances in Halides and Chalcogenides, *Acta Crystallogr. Sect. A: Cryst. Phys., Diffr., Theor. Gen. Crystallogr.* **1976**, *32* (5), 751–767.
